# Re-evaluation of the cardioprotective effects of cannabinoids against ischemia-reperfusion injury according to the IMproving Preclinical Assessment of Cardioprotective Therapies (IMPACT) criteria

**DOI:** 10.3389/fphar.2024.1382995

**Published:** 2024-05-30

**Authors:** Anna Pędzińska-Betiuk, Eberhard Schlicker, Jolanta Weresa, Barbara Malinowska

**Affiliations:** ^1^ Department of Experimental Physiology and Pathophysiology, Medical University of Bialystok, Bialystok, Poland; ^2^ Department of Pharmacology and Toxicology, University of Bonn, Bonn, Germany

**Keywords:** Cannabinoids, Cannabinoid receptor, Cannabidiol, Myocardial ischemia-reperfusion injury, Myocardial infarction, IMproving Preclinical assessment of Cardioprotective therapies (IMPACT)

## Abstract

Ischemic heart disease, associated with high morbidity and mortality, represents a major challenge for the development of drug-based strategies to improve its prognosis. Results of pre-clinical studies suggest that agonists of cannabinoid CB_2_ receptors and multitarget cannabidiol might be potential cardioprotective strategies against ischemia-reperfusion injury. The aim of our study was to re-evaluate the cardioprotective effects of cannabinoids against ischemia-reperfusion injury according to the IMproving Preclinical Assessment of Cardioprotective Therapies (IMPACT) criteria published recently by the European Union (EU) CARDIOPROTECTION COST ACTION. To meet the minimum criteria of those guidelines, experiments should be performed (i) on healthy small animals subjected to ischemia with reperfusion lasting for at least 2 hours and (ii) confirmed in small animals with comorbidities and co-medications and (iii) in large animals. Our analysis revealed that the publications regarding cardioprotective effects of CB_2_ receptor agonists and cannabidiol did not meet all three strict steps of IMPACT. Thus, additional experiments are needed to confirm the cardioprotective activities of (endo)cannabinoids mainly on small animals with comorbidities and on large animals. Moreover, our publication underlines the significance of the IMPACT criteria for a proper planning of preclinical experiments regarding cardiac ischemia-reperfusion injury.

## 1 Introduction

The energy supply of the heart is very high and mainly depends on aerobic metabolism. The high oxygen consumption is further increased by rises in heart rate, contractility and ventricular wall. For this reason, a proper maintenance of the balance between oxygen supply and consumption is extremely important; changes in this balance may result in ischemia (Boyette and Manna, 2023). Sudden myocardial ischemia (I) is usually caused by the acute rupture of an atherosclerotic plaque and the obstruction of a coronary artery leading to an acute coronary syndrome and acute myocardial infarction (AMI) (Basalay et al., 2020). When blood flow and oxygen delivery is limited, anaerobic metabolic processes will be activated finally resulting in damage to the cardiac tissue. Reperfusion (R), which restores the blood supply to the cardiac muscle, halts the progression of myocardial ischemic injury but simultaneously produces sublethal to lethal reperfusion injury in marginally viable myocardium aggravating its damage. In consequence, both events lead to the myocardial ischemia/reperfusion (I/R) injury (Buja, 2023; Wang et al., 2023). Multiple mechanisms are involved in the pathogenesis of the ischemic and reperfusion period including changes in cell metabolism, impairment of mitochondrial function, enhanced inflammatory response, disability of autophagy, platelet-dependent activation of leukocytes, escalating overproduction of reactive oxygen species (ROS) and intracellular calcium overload (Kalogeris et al., 2016; Buja, 2023; Ferdinandy et al., 2023; Liu et al., 2023).

## 2 Cannabinoids and the heart

**FIGURE 1:**
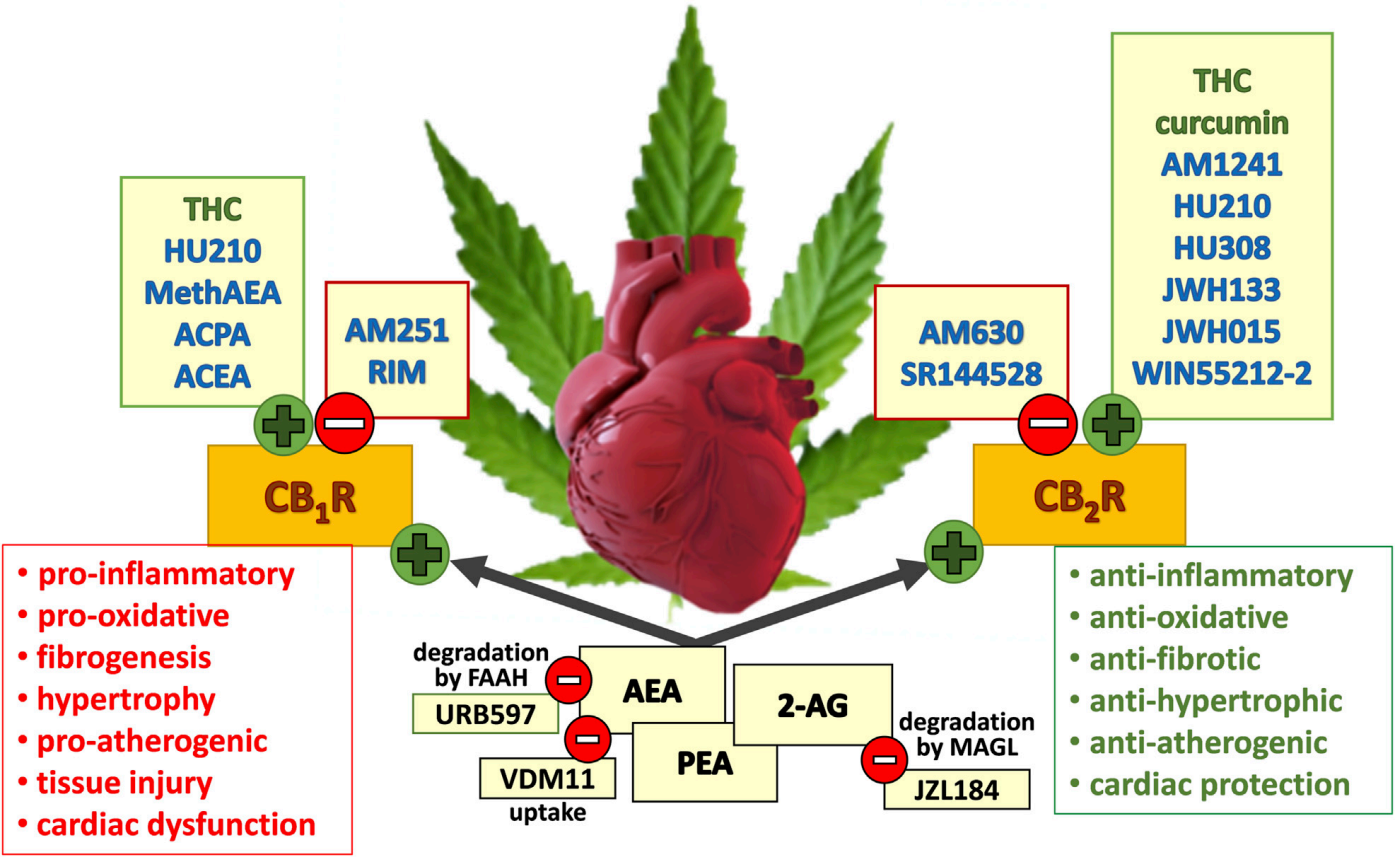
Potential beneficial and detrimental effects of the (endo)cannabinoids considered in this review and mediated via CB_1_R (cannabinoid receptor type 1) and CB_2_R activation (cannabinoid receptor type 2). Green circles with a plus sign describe (partial) agonism at the respective receptor; red circles with a minus sign describe antagonism, inverse agonism or inhibition at the respective mechanism (degradation or uptake). Synthetic, plant-derived compounds and endocannabinoids are written in blue, green, and black font, respectively. 2-AG, 2-arachidonoylglycerol; ACEA, arachidonyl-2′-chloroethylamide; ACPA, arachidonylcyclopropylamide; AEA, anandamide; FAAH, fatty acid amide hydrolase; MAGL, monoacylglycerol lipase; MethAEA, methanandamide; PEA, N-palmitoylethanolamide; RIM, rimonabant; THC, Δ^9^-tetrahydrocannabinol. The scheme is based on figures shown by Pacher and Haskó 2008; Steffens and Pacher 2012; Tang et al., 2021; Weresa et al., 2022; Rorabaugh et al., 2023; Maccarrone et al., 2023.

Numerous compounds including cannabinoids have been suggested for protection of the heart against I/R injury. Cannabinoids can be divided into three groups, i.e., (i) phytocannabinoids found in the cannabis plants *Cannabis sativa or indica*, including psychoactive Δ^9^-tetrahydrocannabinol (∆^9^-THC) and non-psychoactive cannabidiol (CBD), (ii) synthetic cannabinoids, e.g., WIN55212-2 and HU-210 and (iii) endocannabinoids (ECBs), such as anandamide (AEA) and 2-arachidonoylglycerol (2-AG). Removal of the endocannabinoids from the biophase occurs by degradation (e.g., by fatty acid amide hydrolase, FAAH, and monoacylglycerol lipase, MAGL) and cannabinoid reuptake [Figure 1, (Toczek and Malinowska, 2018; Maccarrone et al., 2023),]. Cannabinoids act, although with strongly differing affinity, via the G protein-coupled classical cannabinoid receptors (CBRs) CB_1_ and CB_2_ (Figure 1), some G protein-coupled orphan receptors (GPRs, e.g., GPR55), transient receptor potential vanilloid 1 (TRPV1) receptors and nuclear peroxisome proliferator-activated receptors (PPARs).

Gene and/or protein expression of CB_1_Rs and CB_2_Rs has been detected in the left human ventricle, right atrium, coronary artery endothelial and smooth muscle cells and epicardial adipose tissue. Cannabinoid receptors have also been identified in hearts of the guinea pig and in the left ventricle and left atrium of rat and mouse (Rajesh et al., 2010; 2022; Szekeres et al., 2018; Weresa et al., 2022). CB_1_Rs are localized on arterial and capillary endothelial cells, whereas CB_2_Rs are found on cardiomyocytes and endothelial cells of larger arteries (Lépicier et al., 2007). Moreover, presynaptic CB_1_Rs are also present on sympathetic nerve endings innervating human (Molderings et al., 1999) and rat (Ishac et al., 1996) heart. CB_2_Rs are also localized in rodent myocardial fibroblasts, B cells, and T cells (Defer et al., 2009; Puhl, 2020). CB_1_Rs were also identified in mice cardiac fibroblasts (Valenta et al., 2018) although their function has not been evaluated.

Depending on the type of cannabinoid receptor, its stimulation causes opposite effects (Pacher et al., 2018; Rorabaugh et al., 2023). Thus, CB_1_R activation induces a negative inotropic effect (also in humans: Bonz et al., 2003; Sterin-Borda et al., 2005), decreases noradrenaline release from the cardiac sympathetic nerve endings (Ishac et al., 1996; Molderings et al., 1999), leads to vasodilatation of rat coronary artery (e.g., Szekeres et al., 2018), stimulates oxidative stress and cell death in human endothelial cells and cardiomyocytes (Rajesh et al., 2010), increases smooth muscle proliferation and promotes vascular inflammation, atherosclerosis and cardiac injury (Pacher et al., 2018). On the other hand, CB_2_R stimulation produces a positive inotropic effect (e.g., Sterin-Borda et al., 2005), decreases the acute inflammatory response and consequent oxidative stress in immune and activated endothelial cells (Rajesh et al., 2022) and reduces cardiac fibrosis by diminishing fibroblast-myoblast transformation and collagen production (Defer et al., 2009).

Proper cannabinoid signalling might play a protective role against cardiac injury caused by an imbalance in oxygen supply and demand. As shown in Figure 1, activation of CB_1_Rs leads to deleterious consequences resulting from enhancement in oxidative stress, immune cell migration, inflammation, proliferation and fibrosis. On the other hand, CB_2_ receptor activation mainly has a beneficial effect by an influence opposite in direction to that of the CB_1_Rs (Pacher et al., 2018; Smoum et al., 2022). However, despite previously observed cardioprotective effects mediated mainly via CB_2_ receptor activation [for review, see (Lamontagne et al., 2006; Pacher and Haskó, 2008; Steffens and Pacher, 2012; Maslov et al., 2016; Tang et al., 2021; Rorabaugh et al., 2023; More et al., 2024)] the number of studies reporting cardioprotective effects of cannabinoids in different cardiac disorders seems to decline. The previous studies are not even included in the most recent analysis of multitarget strategies proposed to reduce myocardial I/R injury (Davidson et al., 2019; Heusch, 2023). For better understanding the obstacles for the translation from successful preclinical studies on cardioprotection to clinical practice, the COST ACTION cardioprotection consortium proposed an *in vivo* set of step-by-step criteria. This approach is termed IMproving Preclinical Assessment of Cardioprotective Therapies (IMPACT) and its aim is to improve the likelihood of translating novel cardioprotective interventions into the clinical setting for patient benefit [(Lecour et al., 2021); for details, see below]. Although the IMPACT criteria primarily consider infarct size in experiments *in vivo*, we decided to extend our analysis to other effects, such as inflammation or cardiac contractility to assay the possible broad spectrum of cardiac effects of cannabinoids.

## 3 Aims of the study and methodological approach

The *first* aim of this review is to summarize the current knowledge about the potential cardioprotective role of the (endo)cannabinoid system under hypoxia, ischemia and reperfusion. Data from humans with coronary heart disease (mainly determinations of endocannabinoid levels) are described first (Table 1), followed by animal studies in which the putative cardioprotective role of cannabinoids in ischemic preconditioning is considered (Table 2). Then, we describe *in vivo* experiments on animals exposed to temporary (I/R studies) or permanent occlusion (acute myocardial infarction studies) of the left descending artery (LAD) (Tables 3, 4) and *in vitro* experiments on isolated cardiomyocytes undergoing hypoxia and on isolated hearts subjected to LAD occlusion, low-flow or global no-flow (Table 5). In the second part of the review, we evaluate whether the *in vivo* models and experimental protocols considered here (Tables 3, 4) fulfil the IMPACT criteria and allow translation of the cardioprotective effects of cannabinoids in I/R injury to clinical settings.

**Table 1:** Influence of acute and chronic cardiac disorders on the endocannabinoid system in humans.

Cardiac disease of patients/size of sample	Effects of cardiac disease on ECB levels and cardiac CBR expression (in comparison to the control)^1^	Authors' final conclusions and suggested cardioprotective mechanisms	Ref.
AMI (23 AMI and 16 control patients)	↑2-AG level in blood samples from infarct-related coronary artery but not from radial artery	ECS is activated in AMI and may exert beneficial effects	Wang et al. (2012)
	↔AEA level in blood samples from coronary (including infarct-related) and radial arteries		
	↑serum levels of ROS and TNF-α in infarct-related coronary artery and radial artery		
(43 AMI patients)	↑AEA level in the infarct-related coronary artery compared to the aortic root		Maeda et al. (2009)
	2-AG undetectable (no control patients)		
Stable effort angina (6 patients)	no detectable AEA and 2-AG level in stenotic lesion (no control patients)		Maeda et al. (2009)
CAD (48 patients)	↑arterial blood levels of 2-AG; considerably higher level in coronary arteries than in peripheral arteries and in NSTEMI than in CAD patients	ECB levels vary locally and peak at sites of vascular inflammation	Jehle et al. (2019)
NSTEMI (15 patients)	↑AEA, PEA and AA levels: comparable elevations in all groups		
	2-AG levels correlated with peak troponin and IL-6 levels		
CAD (20 CAD and 20 control patients)	higher venous blood levels of AEA and 2-AG in patients with CAD compared to patients without CAD	ECS is activated in CAD with ↑levels of blood ECBs and ↑expression of CB_1_Rs in coronary atheroma	Sugamura et al. (2009)
	mRNA for CB_1_Rs in coronary atherectomy samples: higher in patients with unstable angina than in those with stable angina		
Chronic heart failure (12 CHF and 12 control patients)	↑blood levels of AEA and 2-AG	ECS may be involved in the regulation of chronic heart failure	Weis et al. (2010)
	↓cardiac CB_1_Rs and ↑CB_2_Rs		
Heart failure of ischemic etiology (24 CHF and 15 control patients)	in ischemic tissue:	ECS may be a modulator of ischemic heart failure since, in patients with altered lipid profile, alterations in the levels of 2-AG and AEA and of MAGL activity were observed in the left ventricle	van Esbroeck et al. (2020)
	↑CB_1_R expression (in half of the ischemic samples), ↔ CB_2_R expression		
	↓DAGLβ, MAGL and ABHD6		
	only in subgroup with altered lipid profile:		
	↑AEA, ↓ 2-AG		
	↑N-acylethanolamine and free fatty acids		
	↓↓MAGL activity (with no differences in protein staining)		

↑, increase; ↓, decrease; ↔, no effect;

1 if not stated otherwise

Abbreviations: 2-AG, 2-arachidonoylglycerol; AA, arachidonic acid; ABHD6, α,β-hydrolase domain containing proteins 6 and 12; AEA, anandamide; AMI, acute myocardial infarction; CAD, coronary artery disease; CB_1_R, CB_2_R, cannabinoid CB_1_/CB_2_ receptor; CBR, cannabinoid receptor; CHF, chronic heart failure; DAGLβ, diacylglycerol lipase β; ECBs, endocannabinoids; ECS, endocannabinoid system; IL-6, interleukin 6; MAGL, monoacylglycerol lipase; NSTEMI, non-ST-elevation myocardial infarction; PEA, palmitoylethanolamide; ROS, reactive oxygen species; TNF-α, tumor necrosis factor-α.

**Table 2:** Cardioprotective role of endocannabinoids in various preconditioning strategies *in vivo* and *in vitro.*

Model/species/protocol applied for low oxygen conditions	Protocol of preconditioning and of drugs targeting the ECS (concentrations in μM or dosage in mg/kg)	Effects of preconditioning and ECBs and their modification by receptor antagonists or enzyme inhibitors (concentrations in μM or dosage in mg/kg) (in comparison to respective controls)	Authors' final conclusions and suggested cardioprotective mechanisms	Ref.
perfused rat heart: **I**: low flow - 30 min **R**: 20 min	ischemic preconditioning - 10 minutes **before I**: no flow for 5 min - 2-AG (0.3) or PEA (0.3) or AEA (0.01–10) perfused 15 min **before I** to the end of **R**	(i) prevention of the ischemia-induced ↓coronary artery vasodilatation to the endothelium-dependent vasodilator 5-HT (10) but not to the endothelium-independent vasodilator SNP (3) (ii) RIM (1) and SR144528 (1) blocked (a) the beneficial effects of preconditioning and (b) the influence of 2-AG (RIM and SR144528) and PEA (RIM) against endothelial dysfunction in coronary artery following **I** **/** **R** (iii) 2-AG and PEA (but not AEA) mimicked the beneficial effect of ischemic preconditioning	ECBs acting via CB_1_Rs and CB_2_Rs play an important role in the endothelial protective effects of ischemic preconditioning	Bouchard et al. (2003)
rats: **I**: LAD occlusion - 30 min **R**: 120 min	remote preconditioning mesenteric artery occlusion for 15 min ended 15 min **before I**	↓infarct size anti-arrhythmic effects: ↓number and duration of arrhythmias; blocked by AM630 (1) *i.v.* but not by AM251 (1) *i.v.* given 15 min prior to remote preconditioning other effects: neither AM251 nor AM630 was able to prevent **I** **/** **R** induced hypotension	ECBs acting via CB_2_Rs are involved in the cardioprotective phenomenon of remote preconditioning	Hajrasouliha et al. (2008)
rats: **I**: LAD occlusion - 30 min **R**: 24 h	propofol preconditioning propofol (10), bolus *i.v*. + 39 mg/kg/h infusion 1 h **before I** until the end of **I**	↓infarct size; reversed by AM630 (1) *i.v.* but not AM251 (1) *i.v.* given 1.5 h before **I** anti-cardiac injury and anti-oxidative effects: ↓serum cTnI, MDA and MPO levels; attenuated by AM630 (1) *i.v.* but not AM251 (1) *i.v.* other parameters: ↑serum AEA and 2-AG (highest 1 h after **I**)	ECBs acting via CB_2_Rs (but not CB_1_Rs) are involved in the cardioprotective and antioxidative effects of propofol preconditioning	Sun et al. (2017)
neonatal rat ventricle cells: **H**: 12 h **R**: 4 h	propofol preconditioning propofol (10), 1 h **prior** to **H** until the end of **H**; *in some experiments:* URB597 (1) or VDM11 (10) 1.5 h **before H** until the end of propofol preconditioning	↑cardiac AEA and 2-AG at the end of H and (time-dependent) during R ↑CB_1_Rs and CB_2_Rs; ↑FAAH protein levels but ↓FAAH activity anti-cardiac injury and anti-oxidative effects: ↑cell viability, ↓LDH leak, ↓cell apoptosis, ↓ROS, ↓MDA, ↑SOD URB597 and VDM11 mimicked the effects of propofol preconditioning		
perfused rat heart: **I**: global no-flow - 20 min **R**: 120 min	NO-induced delayed preconditioning NO; 0.15 mg/h/kg, transdermal application for 24 or 48 h before heart isolation; 2-AG (1) or noladinether (0.1) 30 min **before I** and **during R**	(i) ↑cardiac 2-AG (but not AEA) (ii) 2-AG and noladinether mimicked the cardioprotective effects of NO-induced preconditioning, ↑+dp/dt and LVDP and ↓infarct size (iii) AM251 (0.3), but not AM630 (0.3) prevented the protective effect of preconditioning on infarct size	ECBs acting via CB_1_Rs are involved in the cardioprotection triggered by NO preconditioning	Wagner et al. (2006)
perfused rat heart: **I**: LAD occlusion - 30 min **R**: 120 min	heat stress (HS) preconditioning HS 15 min, 24 h before heart isolation; RIM (1) or SR144528 (1) 5 min **before I** and **during I**	↓infarct size; abolished both by SR144528 (1) and L-NAME (3) but not by RIM (1); RIM, SR144528 or L-NAME: ↔infarct size and risk zone in non-heat stress hearts	ECBs, acting via CB_2_Rs and NO, exert the cardioprotective effect conferred by heat stress preconditioning	Joyeux et al. (2002)
perfused rat heart: **I**: low flow - 90 min **R**: 60 min	LPS preconditioning LPS (10) *i.v.* 24 h before heart isolation; 24 h RIM (1) or SR144528 (1) 5 min **before I** and **during I**	↓infarct size and improved cardiac contractility; abolished both by SR144528 and NNLA (3) but not by RIM SNP (1)-induced ↓infarct size abolished by SR144528 but not RIM RIM, SR144528 or NNLA: ↔infarct size and risk zone in hearts from rats not treated with LPS	ECBs, acting via CB_2_Rs and NO, are involved in the cardioprotection triggered by LPS preconditioning	Lamontagne et al. (2006)

For explanation of the ligands targeting the endocannabinoid system, see Figure 1. ↑, increase; ↓, decrease; ↔, no change.

Abbreviations: 2-AG, 2-arachidonoylglycerol; 5-HT, 5-hydroxytryptamine, serotonin; +dp/dt, the maximum of the first derivative of left ventricular pressure; AEA, anandamide; CB_1_R, CB_2_R, cannabinoid CB_1_/CB_2_ receptor; cTnI, cardiac troponin I; ECBs, endocannabinoids; ECS, endocannabinoid system; FAAH, fatty acid amidohydrolase; H, hypoxia; H/R, hypoxia/reoxygenation on isolated cells; HS, heat stress; ischemia; I/R, ischemia/reperfusion; *i.v.,* intravenous; LAD, left anterior descending artery; LDH, lactate dehydrogenase; L-NAME, N(G)-nitro-L-arginine methyl ester; LPS, lipopolysaccharide; LVDP, left ventricular developed pressure; MDA, malondialdehyde; MPO, myeloperoxidase; NNLA, N-nitro-L-arginine; NO, nitric oxide; PEA, palmitoylethanolamide; R, reperfusion; RIM, rimonabant; ROS, reactive oxygen species; SNP, sodium nitroprusside; SOD, superoxide dismutase.

**Table 3:** Cannabinoid CB_2_R-based cardioprotective actions of cannabinoids in *in vivo* models of ischemia/reperfusion and acute myocardial infarction.

Species, model (protocols of I/R or AMI)	Cannabinoid receptor agonist^1^ (dosage in mg/kg) or receptor deletion; experimental protocol	Effects of agonists^1^ or receptor deletion and their modification by cannabinoid receptor antagonists (dosage in mg/kg) (in comparison to respective controls)	Authors' final conclusions and suggested CB_2_R-based cardioprotective mechanisms against I/R or AMI	Ref.
ISCHEMIA/REPERFUSION				
rat: **I**: LAD occlusion - 30 min **R**: 120 min	AM1241 (3 or 6) once i.p. 5 min **before I**	↓infarct size, ↓area at risk (dose-dependent effects) anti-fibrotic effects: ↓myocardial fibrosis rate anti-inflammatory effects: ↓inflammatory cell infiltration pro-autophagy: cardiomyocyte levels of ↑Pink1, Parkin and Beclin-1, ↑p62 protein, ↑LC3-II/LC3-I ratio anti-apoptotic effects: ↓TUNEL-positive cells anti-myocardial injury: ↓serum cTnI, CK-MB, AST and LDH levels other effects: ↑cardiac CB_2_Rs	cardioprotective effects by induction of autophagy via the Pink1/Parkin pathway	Liu et al. (2021)
rat: **I**: LAD occlusion - 30 min **R**: 120 min	JWH133 (20), once i.v. 5 min **before I**	↓infarct size, ↔ area at risk anti-apoptotic effects: ↓apoptotic index, ↓cardiac cleaved caspases-3 and -9 mitochondrial protection: ↓release of mitochondrial cytochrome c to the cytosol, ↓loss of mitochondrial membrane potential other effects: ↑cardiac p-Akt AM630 (1) i.v. blocked all above effects	cardioprotective effects by prevention of apoptosis (mediated by mitochondrial protection and PI3K/Akt pathway)	Li et al. (2013b)
mice: **I**: LAD occlusion - 30 min **R**: 8 or 24 h	JWH133 (20), once i.p. 5 min **before R**	↓infarct size, ↔ area at risk anti-inflammatory and anti-oxidative effects: ↓superoxide, ↓oxidative stress and neutrophil infiltration (no influence on neutrophil chemoattractants, ↔ TNF-α, CXCL1, CXCL2, CCL3, and ICAM-1) anti-myocardial injury: ↓serum levels of cTnI (only after 1h) other effects: ↑ERK-1 (but not ERK-2), ↑STAT-3, ↔p-Akt, ↓cardiac CB_2_R mRNA level after I/R AM630 (1) i.p. blocked changes in infarct size and cTnI	reduction of infarct size through reduced superoxide generation and neutrophil recruitment and increased ERK-1/2 and STAT-3 phosphorylation	Montecucco et al. (2009)
mice: **I**: LAD occlusion - 30 min **R**: 24 h	HU308 (2), once i.p. 1 h **before I**	↓infarct size anti-inflammatory and anti-oxidative effects: ↓serum levels of ROS and TNF-α other effects: after 24 h: slight ↑serum 2-AG, ↑AEA, ↑cardiac CB1Rs and CB_2_Rs AM630 (2, i.p.) blocked all above effects of HU308	strong cardioprotective effect by ECBs released during **I**/**R**	Wang et al. (2012)
anesthetized mice: **I**: LAD occlusion - 25 min **R**: 120 min	WIN55,212-2 (3.5) or AM630 (1) or AM251 (3), once i.p. 30 min **before I**	WIN55,212-2: ↓infarct size, ↔area at risk anti-inflammatory and anti-oxidative effects: ↓leukocyte-dependent myocardial damage ↓cardiac CXCL8 and IL-1, cardiac MPO activity ↔ number of circulating PMN and lymphocytes ↔ CD11b on blood neutrophils AM630 (but not AM251): slightly ↑infarct size, ↑IL-1 and CXCL8, ↑cardiac MPO and almost abolished cardioprotective effect of WIN55212-2	CB_2_R activation by exogenous and endogenous cannabinoids reduces the leukocyte-dependent myocardial damage	Di Filippo et al. (2004)
Zucker diabetic fatty rat**:** perfused heart **I**: global no-flow - 45 min **R**: 60 min	WIN55,212-2 (1), once i.p. 40 min **before** heart removal and 70 min **before I**	improvement in cardiac work: restored CPP and HR; blocked by AM630 (1) i.p. but not AM251 (1) i.p. other effects: ↓cardiac iNOS and ↑eNOS expression; not blocked by AM630 and AM251	CB_2_Rs and the restoration of the iNOS/eNOS cardiac equilibrium (mediated probably by targets distinct from CBRs) are responsible for cardioprotection in an experimental model of metabolic disorder	González et al. (2011)
rat: **I**: LAD occlusion - 30 min **R**: 120 min	AEA (1), i.v. once, 24 h **before I**	↓infarct size, ↔area at risk anti-myocardial injury: ↑HSP72 AEA effects abolished by AM630 (1) i.v., PI3K inhibitor wortmannin (0.00015) i.v., Akt inhibitor MK-2206 (0.3) i.v. but not by AM251 (1) i.v.	cardioprotection by AEA involving HSP72 and the PI3K/Akt signaling pathway	Li et al. (2013a)
WT and CB_2_ˉ^/^ˉ mice: **I**: LAD occlusion - 60 min **R**: 1, 3 days or 4 weeks	CB_2_ˉ/ˉ JWH133 (3), once i.v. 5 min **before R**	CB_2_ˉ/ˉ vs WT: after 1 day: ↑infarct size after 3 days: ↑cardiac myocyte loss, ↑proapoptotic cleaved caspases-3, ↑cell infiltration of macrophages, ↑remodelling response (↑α-SMA-positive myofibroblasts) after 4 weeks: ↑TGF-β1, ↑collagen 1 and 3 mRNA in hearts, ↑fibrotic scar, ↑LV dilation, ↓EF aged (25 week-old) CB_2_ˉ/ˉ mice developed myocardial hypertrophy only in WT not in CB_2_ˉ/ˉ: 1 day after JWH133: ↓infarcted area	ECB-related cardioprotective effect in post-**I**/**R** cardiac remodeling (cardiomyopathy), potentially via activation of antiapoptotic, prosurvival, and antifibrogenic pathways	Defer et al. (2009)
WT and CB_2_ˉ^/^ˉ mice: **I**: LAD occlusion - 15 min **R**: 1, 3, 5 or 7 days	CB_2_ˉ/ˉ	CB_2_ˉ/ˉ vs WT: after 3 days: ↓induction of anti-oxidative enzymes and contractile elements (lack of ability to induce a switch in myosin heavy chain isoforms), ↑macrophage infiltration, ↑cardiac ROS, ↑collagen deposition, ↑apoptosis (caspase 3), impaired anti-inflammatory response (cardiac ↓IL-10, CCL2, ↓TNF-α), ↑CB_2_Rs (only WT) in hearts after 7 days: ↑microinfarctions, ↑cardiomyocyte loss, ↑anterior wall thickness, ↑AEA and arachidonic acid, ↔2-AG in hearts after 60 days: only partial regression of fibrosis	ECB-related cardioprotective effect after ischemia via ↑contractility, ↓oxidative stress, ↓inflammation, ↓apoptosis, proper formation of infarction border zone, ↓collagen deposition and organization of a stable scar during remodeling	Duerr et al. (2014)
	
WT and CB_2_ˉ^/^ˉ mice: **I**: LAD occlusion - 60 min **R**: 6 h, 1, 3 or 7 days	CB_2_ˉ/ˉ	CB_2_ˉ/ˉ vs WT: after 6 h up to 7 days: ↓induction of cardiac anti-oxidative enzymes and contractile elements (lack of ability to induce a switch in myosin heavy chain isoforms), ↑neutrophils in ischemic area, ↑macrophage infiltration after 1 day: cardiac ↑IL-1β protein, ↔TNF-α mRNA after 3 days: ↓phagocytosis after 7 days: non-compacted scar formation, ↑cardiac collagen deposition, ↓EF and ↑end-diastolic volume, ↓stroke work no compensatory activation of ECBs after I (in contrast to WT)		Duerr et al. (2015)
ACUTE MYOCARDIAL INFARCTION				
mice: LAD occlusion, final effects determined 6 h or 7 days later	JWH133 (1, 3, or 10), once i.p. or HU308 (2), once i.p. 5 min **before AMI**	JWH133 (dose-dependent effects): ↓infarct size (dose-dependent effect, 6 h and 7 days after AMI) improvement in cardiac work: ↑EF, ↑FS, ↓LVESD, and ↓LVEDD (7 days after AMI) anti-myocardial injury: ↓serum levels of CK-MB and LDH (6 h after AMI) JWH133 and HU308: anti-inflammatory effects: ↓initiation and activation of the cardiac NLRP3 inflammasome, ↓serum IL-1β, IL-18, IFN-γ, TNF-α AM630 (2), i.p. attenuated the anti-inflammatory effects of JWH133	cardioprotective effect linked with anti-inflammatory action in AMI through the inhibition of the NLRP3 inflammasome	Yu et al. (2019)
anesthetized mice**:** LAD occlusion, final effects determined 24 h later	HU308 (2), once i.p. 1 h **before AMI**	↓infarct size anti-inflammatory and anti-oxidative effects: ↓serum levels of ROS, TNF-α other effects: after 24 h: ↑↑serum 2-AG, ↑AEA, ↑expression of cardiac CB_1_Rs and CB_2_Rs AM630 (2) i.p. blocked effects of HU308 on infarct size, ROS and TNF-α	ECBs released during AMI exert a strong cardioprotective effect	Wang et al. (2012)
mice: LAD occlusion, final effects determined 7-28 days later	AM1241 (20/day), i.p. for 7 days **after AMI**	after 14 days: improvement in cardiac work, ↑EF, FS after 28 days: ↔infarct size, ↓fibrosis improvement in myocardial repair and cardiomyocyte proliferation: ↑markers of cardiac progenitor/stem cells (c-kit+ and Runx1+; ki67+) anti-inflammatory & oxidative effects: serum ↓MDA, TNF-α, IL-6, ↑Nrf2 improvement in cardiac work: ↑EF, FS in ischemic hearts anti-fibrotic effects: ↓fibrosis area, cardiac ↓collagen I, collagen III, fibronectin, ↓PAI-1, TIMP-1 other effects: ↑cardiac p-Akt	cardioprotective effect by enhancement of cardiac functional recovery and by anti-fibrotic, anti-inflammatory and anti-oxidative effects via modulation of the PI3K/Akt/Nrf2 pathway	Li et al. (2016)
				Wang et al. (2014)
mice with diabetes mellitus: (high-fat diet; streptozotocin-induced diabetes; AMI induced by two high doses of isoproterenol at 24 h intervals)	curcumin (0, 100 or 200 mg/kg/day, orally) for 19 days **before** and for two days **after AMI**	curcumin (dose-dependent effects): anti-myocardial injury: ↑CK-MB and LDH in myocardium (↑integrity of cardiomyocyte membrane), ↓necrosis of muscle fibres with inflammatory cell infiltration, ↓cardiac tissue oedema anti-oxidative effects: ↓lipid peroxidation and oxidative stress anti-inflammatory effects: ↓TNF-α, IL-1β and IL-6 other effects: ↑body weight, ↓AST and SGPT in serum, prevention of ST segment elevation, glucose level restoration and lipid profile normalization AM630 (1/kg/day, 1 h prior to curcumin for 19 days) i.p. attenuated the effects of curcumin	curcumin exerts a cardioprotective effect against AMI under diabetic conditions	Pawar et al. (2022)
WT and CB_2_ ˉ^/^ˉ mice: LAD occlusion, final effects determined 24 h - up to 21 days later	CB_2_ˉ/ˉ	CB_2_ˉ/ˉ vs. WT: baseline: ↓bone marrow CXCL12 and VCAM-1 expression levels; ↓rise in blood neutrophil and monocyte counts after 2-AG administration (10 mg/kg, i.v.) after 24 h: no difference in reversed pattern of enzyme mRNA levels ↓DAGL, ↑MAGL (bone marrow), ↑DAGL, ↓MAGL (heart); ↑ cardiac and plasma levels of 2-AG with no changes in AEA, PEA, OEA (measured only in WT)	1. altered 2-AG signalling affects leukocyte counts at baseline and after AMI via CB_2_Rs	Schloss et al. (2019)
			2. pharmacological MAGL-inhibition worsens cardiac function after AMI due to enhancement of leukocyte recruitment from bone marrow and inflammation	
mice: LAD occlusion, final effects determined 24 h up to 21 days later	JZL184 (16) i.p. (chronic, 24 h **before AMI** and subsequently every 48 h up to 21 days)	JZL184 after 24 h: ↑infarct size, ↑ventricular rupture, ↑mortality (slightly) pro-inflammatory effects in blood: ↑ granulocytes, ↑ monocytes; in hearts: ↑ neutrophils and monocytes, ↑neutrophil recruiting chemokines CXCL1 and CXCL2, ↑ MMP-9, ↔ monocyte recruiting chemokine CCL2; in plasma: ↑ TNF-α, ↔ CXCL1, CXCL2, CCL2 after 7 days: ↑ventricular scars ↓left ventricular anterior wall thickness, ↓density of collagen I, ↔ apoptotic cells after 21 days: ↓cardiac function: ↓EF, ↑end-systolic/end-diastolic volumes		
WT and CB_2_ˉ^/^ˉ mice LAD occlusion, final effects determined 24 h later	CB_2_ˉ/ˉ	CB_2_ˉ/ˉ vs WT: ↑infarct size impaired cardiac work: ↓EF, ↓FS, ↑LVESD, ↑LVEDD deterioration of autophagy process: ↓Beclin-1 and LC3-II/I ratio, ↑p62, ↓AMPK-mTOR-p70S6K signaling pathways in cardiomyocytes	cardioprotective effect after AMI injury by activation of AMPK-mTOR-p70S6K signaling-mediated autophagy	Hu et al. (2019)

For explanation of the ligands targeting cannabinoid receptors (CBRs) and of knockout mice, see Figure 1. 1CBR antagonists mentioned only if their cardiac effects were determined independent of CBR agonists. 1If not stated otherwise, antagonists had no effects by themselves. ↑, increase; ↓, decrease; ↔, no change.

Abbreviations: 2-AG, 2-arachidonoylglycerol; α-SMA, α-smooth muscle actin; AEA, anandamide; Akt, serine/threonine-specific protein kinase; AMI, acute myocardial infarction; AMPK, AMP-activated protein kinase; AST, aspartate transaminase; Beclin-1, autophagy-related marker; CB_1_R, CB_2_R, cannabinoid CB_1_/CB_2_ receptor; CBRs, cannabinoid receptors; CCL2 and CCL3, chemokine (C-C motif) ligand 2 and 3; CD11b, leukocyte adhesion molecule; CK-MB, creatine kinase MB; c-kit, surface marker to identify certain types of hematopoietic progenitors; CPP, coronary perfusion pressure; cTnI, cardiac troponin I; CXCL, C-X-C motif chemokine ligand; DAGL, diacylglycerol lipase; ECBs, endocannabinoids; EF, ejection fraction; eNOS, endothelial NO synthase; ERK, extracellular signal-regulated kinase; FS, fractional shortening; HR, heart rate; HSP72, heat shock protein; I, ischemia; ICAM, intercellular adhesion molecule; IFN-γ, interferon gamma; IL, interleukin; iNOS, inducible NO synthase; i.p., intraperitoneal; I/R, ischemia/reperfusion; i.v., intravenous; ki67, antigen Kiel 67; LAD, left anterior descending artery; LC3-II and LC3-I, microtubule-associated protein light chain 3; LDH, lactate dehydrogenase; LV, left ventricle; LVEDD, left ventricular end-diastolic diameter; LVESD, left ventricular end-systolic diameter; MAGL, monoacylglycerol lipase; MDA, malondialdehyde; MMP-9, matrix metalloproteinase-9; MPO, myeloperoxidase; mTOR, mammalian target of rapamycin; NLRP3, nucleotide-binding oligomerization domain-like receptor family pyrin domain containing 3 inflammasome; Nrf2, nuclear factor erythroid 2-related factor 2; OEA, oleoylethanolamide; p62, autophagy-related marker; p70S6K, 70-kDa ribosomal protein S6 kinase; PAI-1, plasminogen activator inhibitor; p-Akt, phosphorylated Akt; Parkin, cytosolic E3 ubiquitin ligase; PEA, palmitoylethanolamide; Pink1, mitochondrial Ser/Thr protein kinase; PI3K, phosphoinositide-3-kinase; PMN, polymorphonuclear neutrophil; R, reperfusion; ROS, reactive oxygen species; Runx1, surface marker to identify certain types of hematopoietic progenitors; SGPT, serum glutamic pyruvic transaminase; ST segment, section of ECG between end of S wave and beginning of T wave; STAT-3, signal transducer and activator of transcription 3; TGF-β1, transforming growth factor-1β; TIMP-1, tissue inhibitor of metalloprotease; TNF-α, tumor necrosis factor alpha; TUNEL, terminal deoxynucleotidyl transferase dUTP nick end labelling; VCAM-1, vascular cell adhesion molecule 1; WT, wild type.

**Table 4:** Cardioprotective actions of cannabinoids in *in vivo* models of ischemia/reperfusion and acute myocardial infarction by CB_2_R-independent mechanisms.

Species, model (protocols of I/R or AMI)	Cannabinoid receptor agonist^1^ (dosage in mg/kg) or receptor deletion; experimental protocol	Effects of agonists or receptor deletion and modification by antagonists (dosage in mg/kg) (in comparison to respective controls)	Authors' final conclusions and suggested cardioprotective mechanisms against I/R or AMI	Ref.
ISCHEMIA/REPERFUSION				
rat: **I**: LAD occlusion - 45 min **R**: 120 min	HU210 (0.05), once i.v. 15 min **before I**	↔hypoperfused area ↓absolute and relative weights of the necrotic zone	CBRs exert a cardioprotective effect by delaying the formation of necrotic zones	Ugdyzhekova et al. (2002)
rat: **I**: LAD occlusion - 10 min **R**: 10 min	AEA (10), once i.v. or MethAEA (5), once i.v. 10 min **before I**	anti-arrhythmic effect: ↓incidence of ventricular extrasystoles, tachycardia and heart rhythm disturbances, ↑number of rats without arrhythmias L-NAME (50; *i.a.*), and glibenclamide (0.3; *i.v.*) did not modify the anti-arrhythmic effects of agonists	AEA- and MethAEA improve resistance to arrhythmias induced by **I**/**R** independently (1) from NO and (2) from ATP-dependent K^+^ channels	Krylatov et al. (2002)
mice**:** **I**: LAD occlusion - 30 min **R**: 120 min	RIM (10), once i.v. 10 min **before I**	↔infarct size	RIM has no cardioprotective effect after acute treatment (in contrast to chronic treatment, see next line)	Lim et al. (2009)
CB_1_^–^/^–^, CB_1_^+^/^+^ mice, C57BL/6J mice fed high-fat (HFD) or standard diet (STD): **I**: LAD occlusion - 30 min **R**: 120 min	RIM (10/day), i.p. chronically for 7 days **before I**	↓infarct size: all groups except for CB_1_^–^/^–^ ↔risk zones: all groups ↓body weight: all groups except for CB_1_^–^/^–^ ↔MABP and HR: all groups ↔serum and cardiac adiponectin levels: all groups	RIM-induced infarct limitation may involve CB_1_Rs, although not necessarily cardiac CB_1_Rs (see Table 5) and is unrelated to weight loss or altered adiponectin synthesis	Lim et al. (2009)
mice: **I**: LAD occlusion - 45 min **R**: up to 7 days	JZL184 (16), i.p. 24 h **before I** and subsequently every 48 h up to 7 days **during R**	24 h after **I**: ↑cardiac neutrophils, macrophages and monocytes ↑neutrophil recruiting chemokines (CXCL1 and MMP-9) 14-21 days after **I**: ↑ventricular scar ↔stroke volumes, cardiac output, HR	pharmacological MAGL inhibition worsens cardiac work due to enhancement of cardiac leukocyte recruitment and subsequent inflammation	Schlos et al. (2019)
WT and FAAH ^–^/^–^ mice: **I**: LAD occlusion – daily 15 min with subsequent **R** for 3 or 7 repetitive days; effects determined after 7 and 60 days	FAAH^–/–^	FAAH ^–^/^–^ vs WT: 7 days after **I**: ↑fibrosis, ↑cardiomyocyte area, ↑LV hypertrophy, ↓FS, ↓anterior wall thickening the above detrimental effects were reversed by the PPAR-α receptor antagonist GW6471 (1), *i.v.* 60 days after **I**: ↑cellular infiltration in cardiac tissue	the increase in ECBs may have partially detrimental effects on cardiomyocyte survival due to PPAR-α activation	Rajlic et al. (2022)
rat: **I**: LAD occlusion - 30 min **R**: 120 min	CBD (0.05) once i.v. 10 min **before I** or 10 min **before R**	*effects when given before* ***I*** *and* ***R*** *:* ↓infarct size, ↔area at risk, ↔cardiac mast cell degranulation, ↔HR hypotensive effect: ↓MABP *effects when given before* ***I*** *only:* anti-arrhythmic effect:↓ventricular arrhythmias anti-platelet effect:↓collagen-induced platelet aggregation	CBD exerts acute cardioprotection via different mechanisms when given before **I** (↓ventricular arrhythmias and infarct size) or before **R** (↓infarct size only)	Walsh et al. (2010)
rat: **I**: LAD occlusion – 6 min **R**: 6 min	CBD (0.05), once i.v. 10 minutes **before I**	anti-arrhythmic effect: ↓arrhythmia score, ↓total length of arrhythmias, ↓incidence and duration of ventricular tachycardia all effects were blocked by the adenosine A_1_ receptor antagonist DPCPX (0.1) *i.v.* other effects: ↔QT and QRS intervals, ↔MAPB, ↔HR	CBD protects against **I/R**-induced arrhythmias in an adenosine A_1_ receptor-dependent manner	Gonca and Darıcı, (2015)
rabbit: **I**: LCx occlusion - 90 min **R**: 24 h	CBD (0.1), two injections i.v. 10 min **before I** and 10 min **before R**	↓infarct size anti-myocardial injury: ↑systolic wall thickening, ↑blood flow in the area at risk, ↑cardiac improvement visible in magnetic resonance imaging, ↓plasma cTnI anti-inflammatory effect: ↓cardiac leukocyte infiltration	CBD injection before **I** and again before **R** has cardioprotective effects associated with a reduction in infiltrating neutrophils	Feng et al. (2015)
rat: perfused heart **I**: LAD occlusion - 45 min **R**: 45 min	CBD (5/day), twice i.p. 1 and 24 h **before I**	↔infarct size no influence on cardiac work: ↔LVDP, ↔CF	chronic (but not acute) CBD has a substantial indirect cardioprotective effect mainly by a reduced inflammatory response	Durst et al. (2007)
rat: **I**: LAD occlusion - 30 min **R**: 7 days	CBD (5/day), i.p. 1 h **before I** and every 24 h for 7 days **during R**	↓infarct size and necrotic zone, ↔area at risk improvement in cardiac work: ↔LVEDD, ↑CF anti–inflammatory effect: serum ↓IL-6, ↓inflammatory cell infiltration, ↔TNF-α, ↔CRP, ↔formation of cardiac granulation tissue with early collagen deposition		
rat: **I**: LAD occlusion - 45 min **R**: 48 h	CBD (5/day), chronically i.p. for 10 days **before I**	↓infarct size, ↔area at risk other effects: cardiac expression of AT_2_Rs↑ and ↓AT_1_Rs after **I**/**R**; ↑RISK, PI3K/Akt and MAPK/ERK pathways in heart	CBD given before **I**/**R** has a cardioprotective effect against global and regional **I**/**R** that (in the group with regional **I**/**R**) results from activation of AT_2_Rs and the positive modulation of **R**/**I** rescue kinases (RISK), PI3K/Akt and MAPK/ERK pathways	Franco-Vadillo et al. (2021)
rat: perfused heart **I:** global no-flow - 30 min **R**: 90 min)	CBD (5/day), chronically i.p. for 10 days **before I**	improvement in cardiac work: ↑recovery of +dp/dt and -dp/dt, ↑LVDP, ↑CPP, ↔HR		
ACUTE MYOCARDIAL INFARCTION				
mice: LAD occlusion, final effects determined 24 h later	THC (0.002), single injection i.p. 2 h or 48 h **before AMI**	↓infarct size improvement in cardiac work: ↓ESD, EDD, ↑FS; ↔HR anti-myocardial injury: ↓serum troponin T (2 but not 48 h after THC) ↓(histological) cardiac damage, ↓neutrophil infiltration other effects: ↓phosphorylated ERK (2 but not 48 h after THC)	single and chronic administration of an ultra-low THC dose before AMI reduces myocardial ischemic damage	Waldman et al. (2013)
	THC (0.002), chronically i.p.: 3 injections/week for 3 weeks **before AMI**			
		↓infarct size improvement in cardiac work: ↑FS anti-inflammatory effect: ↓serum TNF-α other effects: ↔ troponin T in the serum, ERK activity in cardiac tissue		
rat: LAD occlusion, final effects determined 12 weeks later	HU210 (0.05/day), i.p. AM251 (0.5/day), i.p. (each drug was administered chronically for 12 weeks, starting 24 h **after AMI**)	HU210 and AM251: ↔infarct size and mortality alteration of cardiovascular work: HU210: ↑LVEDP, ↑MAP, improved CI, SVI; ↓TPRI AM251: stimulation of left-ventricular remodeling indicated by an ↑left-ventricular volume, ↓peak-developed pressure following aortic occlusion, ↓systolic performance other effects: HU210: prevention of endothelial dysfunction in aortic rings and hypotension, ↔ cardiac CB_1_R expression, ↓weight gain and hair loss, ↑anxiety, seizures	after large AMI, a non-selective CBR agonist increases LVEDP, prevents hypotension and aortic endothelial dysfunction but a CB_1_R antagonist promotes remodeling and reduces maximal developed pressure	Wagner et al. (2003)
rats: LAD occlusion, final effects determined 6 weeks later	RIM (10/day), i.p. i. pre-treatment group: started 7 days **before AMI** and continued for 6 weeks ii. post-treatment group: started 3 h **after AMI** and continued for 6 weeks	*i. pre-treatment group*: improvement in cardiac work: ↓heart weight/body weight index ↑+dp/dt, ↑-dp/dt, ↑FS, EF, ↓LVIDs, ↓LVIDd, normalisation of prolonged QRS complex duration, ↓occurrence of lung oedema, ↑SERCA2a anti-fibrotic effects: ↓cardiac and aorta hydroxyproline content *ii. post-treatment group*: ↓LVIDs (7 days but not 6 weeks after AMI) *iii. both groups*: anti-fibrotic effects: ↓mRNA of cardiac TGF-β1, ↓MMP-9 (trend) ↓arterial stiffness 7 days and 6 weeks after AMI other effects: ↓weight gain, ↑motility of the LV anterior and posterior wall after AMI, ↓serum levels of BNP	blocking of CB_1_R improves cardiac functions in the early and late stages after AMI (preventive treatment is even more effective compared to post-ischemic), decreases arterial stiffness and reduces cardiac remodeling	Slavic et al. (2013)
rats: LAD occlusion, final effects determined 6 weeks later	RIM (10), once i.a. 6 weeks **after AMI**)	↑+dp/dt	single administration of RIM 6 weeks after AMI increases the rate of developed LV pressure	Slavic et al. (2013)
WT and GPR55^–^/^–^ mice: LAD occlusion, final effects determined 1 to 28 days later	GPR55^–/–^	GPR55^–^/^–^ vs WT: baseline: bradycardia, ↑body and heart weight, ↑LV mass, ↑LVIDd and LV volume, ↔CO, EF, FS after 1 day: ↔infarct size and myeloid cell infiltration after 3 days: ↑LV wall and septum thickening, ↑LV dilatation ↑pro-repair cardiac macrophage expansion in hearts ↑infarction expansion, prolonged elevation of cardiac ↑IL-1β, CCL2, IL-6 and TNF-α, neutrophils and plasma MPO levels impaired cardiac remodelling: ↑cardiac MMP-9, ↑collagen deposition after 28 days:↓late structural remodelling (↓compensatory hypertrophy of cardiomyocytes)	GPR55 regulates wound healing kinetics, cardiomyocyte hypertrophy and maladaptive remodeling	Puhl et al. (2021)

For explanation of the ligands targeting cannabinoid receptors (CBRs) and endocannabinoid degrading enzymes and of knockout mice, see Figure 1.

^1^CBR antagonists were mentioned only if their cardiac effects wFere determined independent of CBRs agonists. If not stated otherwise, antagonists did not modify cardiac parameters by themselves. I/R and AMI were studied on anaesthetized animals or perfused hearts. ↑, increase; ↓, decrease; ↔, no change.

Abbreviations: +dp/dt, maximum of the first derivative of left ventricular pressure; -dp/dt, minimum of the first derivative of left ventricular pressure; AEA, anandamide; Akt, serine/threonine-specific protein kinase; AMI, acute myocardial infarction; AT_1_R, AT_2_R, angiotensin 1 and 2 receptor; BNP, brain natriuretic peptide; CB_1_R, CB_2_R, cannabinoid CB_1_/CB_2_ receptor; CBRs, cannabinoid receptors; CBD, cannabidiol; CCL2, C-C motif chemokine ligand 2; CF, coronary flow; CI, cardiac index; CO, cardiac output; CPP, coronary perfusion pressure; CRP, C-reactive protein; cTnI, cardiac troponin I; CXCL, C-X-C motif chemokine ligand; DPCPX, 8-cyclopentyl-1,3-dipropylxanthine; ECBs, endocannabinoids; EDD, left-ventricular end-diastolic dimensions; EF, ejection fraction; ERK, extracellular signal-regulated kinase*;* ESD, left ventricular end-systolic diameters; FAAH, fatty acid amidohydrolase; FS, fractional shortening; GPR55, G protein-coupled receptor 55; HR, heart rate; HFD, high-fat diet; I, ischemia; I/R, ischemia/reperfusion; i.a., intraarterial (arteria femoralis); i.p., intraperitoneal; i.v., intravenous; IL, interleukin; LAD, left anterior descending artery; LCx, left circumflex coronary artery; L-NAME, N-nitro-L-arginine methyl ester; LV, left ventricle; LVDP, left ventricular developed pressure; LVEDD, left ventricular end-diastolic diameter; LVEDP, left ventricular end-diastolic pressure; LVIDd, end-diastolic left ventricular diameter; LVIDs, end-systolic left ventricular diameter; MABP, mean arterial blood pressure; MAGL, monoacylglycerol lipase; MAP, mean arterial pressure; MAPK, mitogen-activated protein kinase; MethAEA, methanandamide; MMP-9, matrix metalloproteinase-9; MPO, myeloperoxidase; NO, nitric oxide; PI3K, phosphoinositide 3-kinase; PPAR-α, peroxisome proliferator activated receptor alpha; QRS, sequence of the Q, R and S wave of the electrocardiogram; QT, time from the beginning of the QRS complex to the end of the T wave in the electrocardiogram; R, reperfusion; RIM, rimonabant; RISK, reperfusion injury salvage kinase; SERCA2a, sarcoplasmic/endoplasmic reticulum Ca^2+^-ATPase 2a; STD, standard diet; SVI, stroke volume index; TGF-β1, transforming growth factor β1; THC, Δ^9^-tetrahydrocannabinol; TNF-α, tumor necrosis factor; TPRI, total peripheral resistance index; WT, wild.

**Table 5:** Cardioprotective effects of cannabinoids in different models of low oxygen conditions in isolated cardiac cells and heart preparations.

Model/species/protocols of H/R and I/R	Cannabinoid ligand (concen-trations in μM), receptor deletion and experimental protocol	Effects of agonists or receptor deletion in models of low oxygen conditions and modification by cannabinoid receptor antagonists (concentrations in µM) (in comparison to respective controls)	Authors' final conclusions and suggested cardioprotective mechanisms against hypoxia and ischemia/reperfusion	Ref.
**isolated cardiac cells**				
rat cardiomyocytes (H9c2) **H**: 24 h **R**: 6 h	AM1241 (3) 6 h **before H**	pro-autophagy: ↑autophagy related proteins and markers: ↑Pink1, Parkin and Beclin-1, ↓p62 protein, ↑LC3-II/LC3-I ratio other effects: ↑CB_2_R expression	CB_2_Rs exert cardioprotective effects against **H**/**R** injury by activating Pink1/Parkin-mediated autophagy	Liu et al. (2021)
primary cultured cardiac fibroblasts from the left ventricle of neonatal mice **H**: 12 h	AM1241 (5) **during** the 12 h of **H**	anti-fibrotic effects: ↓collagen I and collagen III, ↓Nrf2 (cytosol), ↑Nrf2 (nucleus), ↓TGF-β1/Smad3 pathway, ↓α-SMA, ↓PAI-1 and TIMP-1 pro-survival parameter: ↑p-Akt/Akt anti-oxidative effects: ↓ROS level, ↑GSH level, ↑SOD activity, ↓MDA content other effects: ↑CB_2_R expression AM630 (1) diminished the AM1241 effects	CB_2_R/Akt/Nrf2 signaling provides cardioprotection via anti-fibrotic and anti-oxidative effects and Nrf2-mediated inhibition of the TGF-β1/Smad3 pathway	Li et al. (2016)
murine primary cardiomyocytes **H**: oxygen-glucose deprivation - 4 h	JWH133 (0.001, 0.01, or 0.1) 10 min **before H**	pro-survival parameters: ↑cell viability, ↓LDH release anti-inflammatory effects: ↓initiation and activation of the NLRP3 inflammasome (the above effects were concentration-dependent)	CB_2_Rs have a cardioprotective effect via inhibition of the NLRP3 inflammasome	Yu et al. (2019)
neonatal rat cardiomyocytes **H**: 100% argon - 1, 1.5 and 2 h	THC (0.1, 1.0 and 10) 24 h **before H**	effects dependent on the duration of **H** pro-survival parameters: ↓LDH release and protection of the distribution of alpha-sarcomeric actin (prevented by SR144528 (10) and L-NAME (100) but not by RIM (10)) NO production: ↑NO and iNOS levels (blocked by SR144528 (10) but not by RIM (10)) other effects: neonatal rat cardiomyocytes express CB_2_Rs but not CB_1_Rs	CB_2_Rs protect cardiac cells by induction of NO production	Shmist et al. (2006)
embryonic cardiomyocytes (eCM) from WT and CB_2_^–^/^–^ mice **H**: different protocols from 6 h to 72 h	CB_2_^–^/^–^	CB_2_^–^/^–^ vs WT: lack of cardiomyocyte protection mechanisms, ↓cardioprotective and antioxidative enzymes HMOX1, GPX-1, mRNA of Rac1 ↓chemokine CCL2 ↔apoptosis, ↔cells lost ↔contractile elements β/α-MHC ratio (↑β-MHC and α-MHC in WT)	CB_2_Rs play a role in adaptation of cardiac contractile elements triggering an inflammatory reaction as an important part of cardioprotective mechanisms	(Duerr et al., 2014, 2015)
embryonic (eCM) and adult (CM) cardiomyocytes from WT and CB_2_^–^/^–^ mice **H**: 24 h	CB_2_^–^/^–^	CB_2_^–^/^–^ vs WT: ↑loss of cardiomyocytes (CM), ↑apoptosis (eCM),↑migration potential of macrophages, ↑aggressive action of macrophages on ischemic cardiomyocytes	CB_2_Rs decrease susceptibility of cardiomyocytes via modification of migration and function of macrophages in interaction with cardiomyocytes, thereby influencing their survival	Heinemann et al. (2015)
primary ventricular cardiomyocytes from WT and CB_2_^–^/^–^ mice **H:** oxygen-glucose deprivation - 6 h	CB_2_^–^/^–^	CB_2_^–^/^–^ vs WT: ↓cell viability autophagy-related proteins: ↓Beclin-1 and LC3-II/I ratio and ↑p62, ↓number of autophagosomes ↑LDH release from cardiomyocytes pro-apoptotic effects: ↑cleaved caspase-3/caspase-3 ratio, ↑Bax protein in cardiomyocytes ↓AMPK-mTOR-p70S6K cardiac protective signaling pathway	CB_2_Rs have cardioprotective effects by activating AMPK-mTOR-p70S6K signaling-mediated autophagy	Hu et al. (2019)
human cardiomyocytes **H**: chemical ischemia^1^, 15 min **R**: 20 min	THC (10) 15 min **before I** and **during R**	pro-survival parameters: ↓cell injury, ↓spherical shape of cardiomyocytes, ↓vacuolisation of cytoplasm and swollen mitochondria, ↓number of dead cells, ↑CERK anti-inflammatory effects: MMP-2 activity restored to the level of the aerobic control, ↔IL-6 anti-oxidative effects: ↑total antioxidant capacity preservation of metabolic function: ↑activity of intracellular acetyl esterase	THC has cardioprotective effects related to improvement in cell metabolism and antioxidative activity, mitochondrial protection and reduced cell mortality (in part probably due to increased CERK activity)	Banaszkiewicz et al. (2022)
rat cardiomyocytes **H**: chemical ischemia^1^, 3 min **R**: 20 min	THC (0.1–10) 15 min **before I** and **during R**	pro-survival parameters: ↑cardiomyocyte contractility, ↑cytoplasmic LDH activity		
murine cardiomyocytes **H,** intermittent: 18 h	AM251 (0.5) **before H** until to its end	anti-apoptotic effect: ↓apoptotic cells anti-oxidative effect: ↓ROS mitochondrial protection: ↓mitochondrial fragmentation, ↑level of mitochondrial membrane potential other effects: ↓CB_1_R expression, ↑AMPK, ↑PGC-1α	CB_1_R blockade can reduce the damage to cardiomyocytes induced by hypoxia via activation of the AMPK/PGC-1α pathway	Hu et al. (2023)
murine atrial cardiomyocytes (HL-1) **H**: 6 h **R**: 18 h	RIM (0.1) for 24 h or for 7 days **before** and **during H/R**	↔cell death HL-1 cells express CB_1_Rs	RIM does not have cardioprotective effects (see Table 4) acting directly on cardiomyocytes or on CB_1_Rs	Lim et al. (2009)
**perfused heart**				
perfused rat heart **I**: LAD occlusion – 30 min **R**: 120 min	JWH133 (0.001, 0.01, or 0.1) for 15 min **before I**	↓infarct size improvement in cardiac work: ↑LVDP, ↑+dp/dt, ↑- dp/dt increase in LVEDP during reperfusion prevented, ↑CF, ↔HR mitochondrial protection: prevention of MTMP opening and prevention of the loss of mitochondrial membrane potential, ↓cytochrome c release from mitochondria other effects: ↑ERK1/2 AM630 abolished the effects of JWH133	the cardioprotective effect of CB_2_Rs against **I**/**R** injury may be through increased ERK1/2 phosphorylation, which inhibits opening of the mitochondrial permeability transition pore	Li et al. (2014)
perfused rat heart **I**: low-flow - 90 min **R**: 60 min	JWH133 (0.01) 15 min **before I** or **during I** or **during R**	↓infarct size (independently on the protocol of JWH133 infusion) ↑functional recovery of +dp/dt (JWH133 perfused before and during **I**) SR144528 (0.1) blocked the beneficial effects of JWH133	CB_2_R activation is able to reduce infarct size when a CB_2_R agonist is administered either before ischemia, during ischemia, or during reperfusion	Lépicier et al. (2006)
perfused rat heart **I**: low flow - 120 min **R**: 20 and 60 min for determination of physiological parameters and infarct size, respectively	PEA (0.3) 2-AG (0.3) AEA (up to 1) ACEA (0.005, 0.05) JWH015 (0.005, 0.05) 15 min **before** and **during I** until the end of **R**	*PEA, 2-AG, ACEA, JWH015*: ↓infarct size *PEA and 2-AG*: improvement in cardiac work and anti-cardiac injury: full recovery in +dp/dt and prevention of the increase in LVEDP during reperfusion, ↓overflow of LDH and CK into the perfusate *PEA*: pro-survival parameters: ↑cardiac p-Akt/Akt, ↑ERK1/2, ↑p38 MAPK phosphorylation level *AEA*: ↔ +dp/dt, LVEDP, LDH and CK effect of PEA and 2-AG completely blocked by SR144528 (1); effect of 2-AG only partially blocked by RIM (1); effect of PEA reduced by the PKC inhibitor chelerythrine (1)	ECBs have strong cardioprotective effects mediated mainly through CB_2_Rs that involve p38 and ERK1/2, as well as PKC activation	Lépicier et al. (2003)
perfused rat heart **I**: low flow - 90 min **R**: 60 min	ACEA (0.05) JWH015 (0.05) 5 min **before** and **during I** until the end of **R**	↓infarct size (ACEA, JWH015) improvement in cardiac work: ↑ventricular contraction (+dp/dt) improved after **R** (JWH015) NO production: ↑cardiac iNOS expression (ACEA) other effects: ↔cardiac CB_1_R expression after **I** and **R**, after **R**: ↓CB_2_Rs (ACEA but not JWH015) RIM (0.1) and SR144528 (0.1) blocked the effects of ACEA and JWH015, respectively NNLA prevented the ability of ACEA (but not JWH015) to reduce the infarct size	both CB_1_Rs (present on endothelial cells and acting via NO production) and CB_2_Rs (present on cardiomyocytes) are involved in cardioprotective effects	Lépicier et al. (2007)
perfused rat heart **I**: global no-flow – 30 min **R**: 120 min	AEA (1) MethAEA (1) ACPA (1) JWH133 (1) ACPA + JWH133 (1, each) 5 min **before** and **during I** until the end of **R**	↓infarct size: AEA and MethAEA ↔infarct size: ACPA and JWH133, individually or combined RIM (1) and SR144528 (1) blocked the infarct-size limiting effect of AEA	cardioprotective effect of AEA in **I**/**R** might involve a new cannabinoid receptor subtype	Undertown et al. (2005)
perfused rat heart **I**: global no-flow - 45 min **R**: 30 min	HU210 (1) RIM (1) SR144528 (1) 10 min **before I**	HU210: ↔HR, ↔LVEDP, transitory ↓+dp/dt and ↓LVDP (disappeared after 5 min) RIM (1) or SR144528 (1): ↔HR, ↔LVEDP	CBRs produce an infarction-limiting effect and simultaneously time-dependently reduce pumping function during R probably associated with a better cardiomyocyte survival	Maslov et al. (2006)
perfused rat heart **I**: LAD occlusion - 45 min **R**: 2 h	HU210 (1) 5 min **before R** and continued for 15 min of **R**	↓infarct size, area at risk changes in cardiac work: ↓LVDP, ↓ product LVDP×HR/1000, ↓HR anti-cardiac injury:↓CK (in perfusate)		Gorbunov et al. (2016)
perfused rat heart **I**: global no-flow - 25 min **R**: 30 min	THC (0.1 - 10) 10 min **before I** and **during** the first 10 min of **R**	improvement in cardiac work: ↑recovery of HR and LVDP, ↑RPP, ↑CF	THC restores heart mechanical function	Banaszkiewicz et al. (2022)
perfused mouse heart (WT and GPR55^–^/^–^) **I**: global no-flow - 30 min **R**: 30 min	GPR55 ^–^/^–^ LPI (10) bolus 10 min **before I** or during 1-2 min of **R**	GPR55^–^/^–^: LPI prior to **I**/**R** or during **R**: ↔infarct size WT: LPI prior to **I**/**R**: ↑infarct size, abolished by ROCK inhibitor Y-27632; LPI during **R**: ↔infarct size	increased LPI levels in the vicinity of a developing infarct may worsen the outcome of AMI during ischemia via activation of GPR55 receptors and via the GPR55 receptor-ROCK/p38-MAPK pathway	Robertson-Gray et al. (2019)

If not stated otherwise, antagonists did not modify cardiac parameters by themselves. ^1^Chemical ischemia was induced by replacing HEPES buffer with ischemia buffer (HEPES buffer containing 2-deoxyglucose and sodium cyanide which served as an inhibitor of the electron transport chain). In the second column, antagonists were entered only if their effects were examined independent from agonists. For explanation of the ligands targeting CBRs, see Figure 1. ↑, increase, ↓, decrease, ↔, no effect.

Abbreviations: α/β MHC, myosin heavy chain alpha/beta; α-SMA, α-smooth muscle actin; +dp/dt, maximum of the first derivative of left ventricular pressure; -dp/dt, minimum of the first derivative of left ventricular pressure; 2-AG, 2-arachidonoylglycerol; ACEA, arachidonyl-2-chloroethylamide; ACPA, arachidonylcyclopropylamide; AEA, anandamide; Akt, serine/threonine-specific protein kinase; AMI, acute myocardial infarction; AMPK, AMP-activated protein kinase; Bax, BCL-2-associated X protein; Beclin-1, autophagy-related marker; CB_1_R, CB_2_R, cannabinoid CB_1_/CB_2_ receptor; CBRs, cannabinoid receptors; CCL2, C-C motif chemokine ligand 2; CERK, ceramide kinase; CF, coronary flow; CK, creatine kinase; CM, cardiomyocyes; ECBs, endocannabinoids; eCM, embryonic cardiomyocytes; ERK, extracellular signal-regulated kinase; GPR55, G protein-coupled receptor 55; GPX-1, glutathione peroxidase 1; GSH, reduced glutathione; H, hypoxia; H/R, hypoxia/reoxygenation on isolated cells; HL-1 cells, murine atrial cardiomyocytes; HMOX-1, heme oxygenase-1; HR, heart rate; I, ischemia; I/R, ischemia/reperfusion; IL, interleukin; iNOS, inducible NO synthase; LAD, left anterior descending artery; LC3-II and LC3-I, microtubule-associated protein light chain 3; LDH, lactate dehydrogenase; L-NAME, N(G)-nitro-L-arginine methyl ester; LPI, L-α-lysophosphatidylinositol; LVDP, left ventricular developed pressure; LVEDP, left ventricular end-diastolic pressure; p38 MAPK, p38 mitogen-activated protein kinase; MDA, malondialdehyde; MethAEA, methanandamide; MMP-2, matrix metalloproteinase-2; MTMP, mitochondrial membrane potential; mTOR, mammalian target of rapamycin; NLRP3, nucleotide-binding oligomerization domain-like receptor family pyrin domain containing 3 inflammasome; NNLA, N-nitro-L-arginine; NO, nitric oxide; Nrf2, nuclear factor erythroid 2-related factor 2; p62, autophagy-related marker; p70S6K, 70-kDa ribosomal protein S6 kinase; PAI-1, plasminogen activator inhibitor; p-Akt, phosphorylated Akt; Parkin, cytosolic E3 ubiquitin ligase; PEA, palmitoylethanolamide; PGC-1α, peroxisome proliferator-activated receptor-gamma coactivator; Pink1, phosphoinositide 3-kinase; PKC, protein kinase C; R, reperfusion; Rac1, ras-related C3 botulinum toxin substrate 1; RIM, rimonabant; ROCK, rho-associated protein kinase; ROS, reactive oxygen species; RPP, rate pressure product expressed as the product of heart rate and left ventricular developed pressure; Smad3, mothers against decapentaplegic homolog 3; SOD, superoxide dismutase; TGF-β1, transforming growth factor β1; THC, Δ^9^-tetrahydrocannabinol; TIMP-1, tissue inhibitor of metalloprotease; WT, wild type.

**FIGURE 2:**
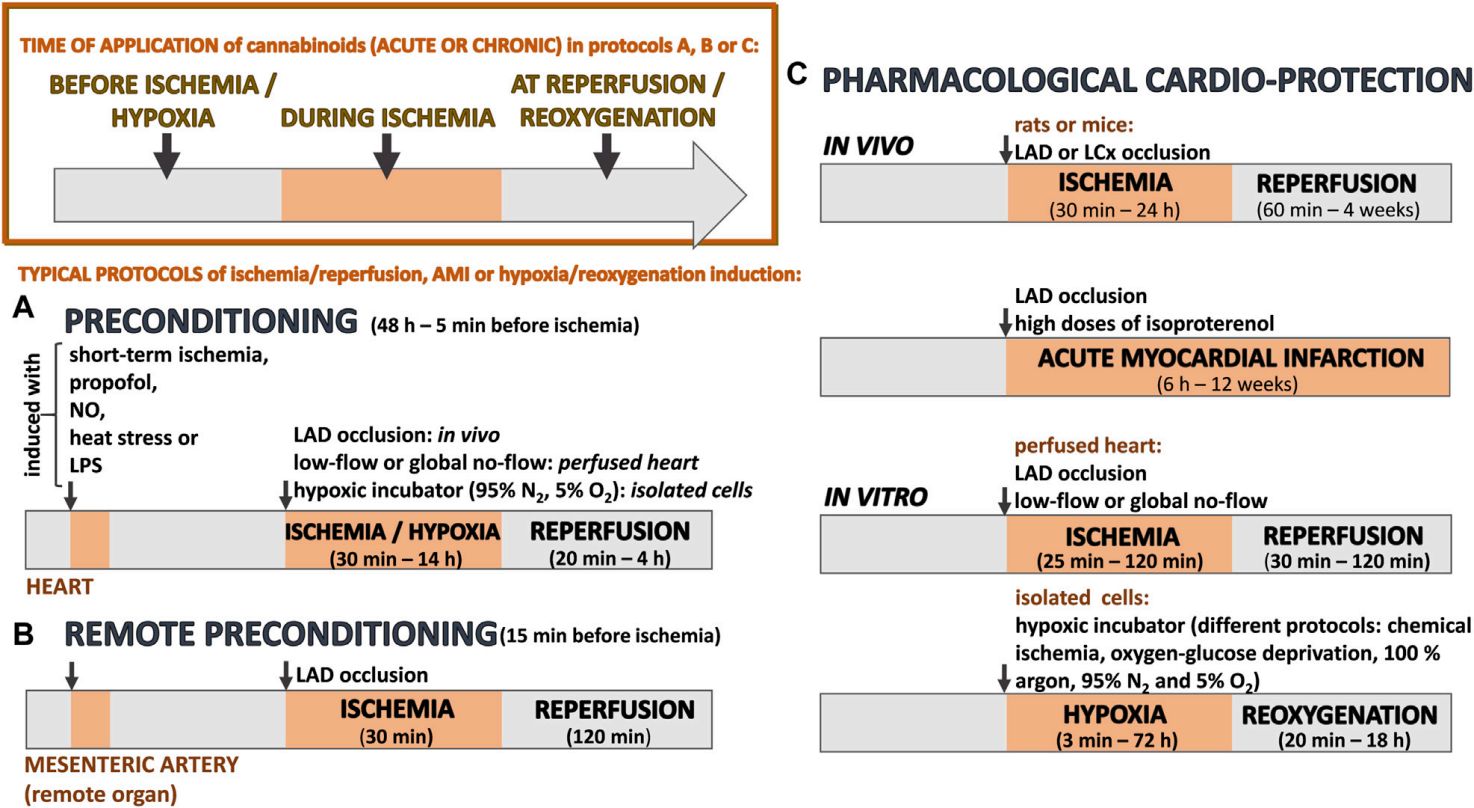
A schematic representation of typical protocols for ischemia, hypoxia or acute myocardial infarction (AMI) that have been used to examine whether (endo)cannabinoids have cardioprotective effects during various preconditioning strategies **(A, B)** or when administered to assess their pharmacological effects **(C)**. At the top of the image (left part), the time of application displays different approaches to administer (endo)cannabinoids. For results, see Tables 1-5. LAD, left anterior descending artery; LCx, left circumflex coronary artery; NO, nitric oxide; LPS, lipopolysaccharide.

To find the relevant articles dealing with the cardioprotective effects of cannabinoids against ischemia-reperfusion injury, we performed a comprehensive search in the PubMed, Medline and EMBASE databases (closed in February 2024). Because there is a substantial amount of research in the databases that deal with ischemia in the nervous system or perinatal hypoxia-ischemia, we added the word “cardiac” to each search. The following key phrases were used: “cardiac ischemia cannabinoid” (which yielded 205 results), “cardiac hypoxia cannabinoid” (21 results), “cardiac preconditioning cannabinoid” (16 results), “human cardiac ischemia cannabinoid” (130 results) and “myocardial infarction cannabinoid” (180 results). Based on the approved therapeutic uses of cannabidiol, search phrases also included “cannabidiol” coupled to “cardiac ischemia”, “cardiac hypoxia” and “myocardial infarction” (a total of 35 results). Titles, abstracts, and full texts of the identified papers were analyzed, and duplicate articles or those with non-relevant content were excluded; only articles in English were considered. In total, 52 publications were included in this review, which are summarized in Table 1-5. A schematic representation of the typical protocols for ischemia, hypoxia or acute myocardial infarction used in the above studies is given in Figure 2.

## 4 Human cardiac disorders modify endocannabinoid levels

The endocannabinoid system is activated during various cardiac disorders in humans (Table 1). Thus, higher endocannabinoid levels (2-AG, AEA or both) were found in patients with coronary artery disease (CAD) and at the ruptured plaque site of the infarct-related coronary artery in patients with AMI (Maeda et al., 2009; Wang et al., 2012) and CAD (Sugamura et al., 2009; Jehle et al., 2019) in comparison to peripheral arteries. Interestingly, the endocannabinoid level was higher in the coronary artery of patients with non-ST-elevation myocardial infarction (NSTEMI) compared to CAD (Jehle et al., 2019) and in the blood of patients with severe chronic heart failure (CHF) in comparison to controls (Weis et al., 2010). Note that, in the latter study, CHF might have been due to ischemia or to other reasons.

As shown in Table 1, there are differences as to which endocannabinoid is enhanced under pathological conditions even within the same disease. Thus, in AMI, the 2-AG level in the infarct-related coronary artery was higher in the paper by Wang et al. (2012) but undetectable in the publication by Maeda et al. (2009). The above discrepancy probably results from methodological differences. For example, Jehle et al. (2019) underlined the influence of the site of blood sampling, i.e., venous (Sugamura et al., 2009) *versus* arterial (Jehle et al., 2019). Of course, one cannot exclude a higher heterogeneity of the experimental groups in humans than in experimental animals. Importantly, as mentioned above, the endocannabinoid levels (AEA and 2-AG) in infarct-related coronary arteries were higher than those in blood samples drawn from distinct locations.

Additionally, in coronary atherectomy samples, CB_1_R mRNA expression was more markedly increased in patients with unstable angina than in those with stable angina (Sugamura et al., 2009). The expression of CB_1_Rs and CB_2_Rs was also studied in human left ventricular myocardium of patients with chronic heart failure (CHF) and controls (Weis et al., 2010). In healthy myocardium, mRNA transcripts of CB_1_Rs and CB_2_Rs were expressed similarly whereas in the myocardium of CHF patients a shift of the CB_1_/CB_2_R ratio towards the expression of CB_2_Rs was observed (Table 1).

An abnormal lipid profile belongs to the risk factors important for cardiovascular disease. Targeted lipidomics analysis showed the existence of two subgroups within the ischemic end-stage failing human left ventricle, the first one resembling controls and the second one presenting with an altered lipid profile. Interestingly, only in the second subgroup decreased 2-AG and increased AEA, N-acylethanolamine and free fatty acids levels as well as a robust reduction in cardiac MAGL activity occurred (van Esbroeck et al., 2020).

The question arises, whether activation of the endocannabinoid system in humans is beneficial or has pathological relevance in myocardial infarction. Unfortunately, we are not able to answer this question based on only few publications related to rather small patient groups (Table 1). Data obtained on cardiac experimental models suggest mainly protective effects of endocannabinoids (see below). On the other hand, an increase in endocannabinoid levels in humans (Table 1) was accompanied by enhanced serum levels of reactive oxygen species (ROS) and tumor necrosis factor-α (TNF-α) in infarct-related coronary artery (Wang et al., 2012) or correlated with peak troponin and IL-6 levels (Jehle et al., 2019) but correlations, of course, do not prove a causal relationship.

## 5 Cardioprotective role of endocannabinoids during preconditioning

Almost four decades ago, Murry and colleagues discovered that brief episodes of ischemia followed by short-lasting reperfusion before a sustained period of coronary artery occlusion reduced infarct size in dogs (Murry et al., 1986). This was the beginning of the development of research on “conditioning” phenomena used to reduce infarct size by brief periods of I/R on the heart. Ischemic preconditioning is considered the gold standard of cardioprotection (Basalay et al., 2020). In remote preconditioning, brief episodes of I/R are administered to organs or tissues other than the heart and can be easily applied in a non-invasive way, e.g., using a blood pressure cuff on an arm or a leg (Lang and Kim, 2022; Penna et al., 2022; Comità et al., 2023; Ferdinandy et al., 2023; Zhao et al., 2023). Another way to protect the heart against the consequences of I/R injury is pharmacological conditioning (i.e., volatile anesthetics, opioids or α_2_-adrenoceptor agonists), a concept that is based on the administration of specific drugs mimicking the effect of ischemic preconditioning (Roth et al., 2021; Penna et al., 2022; Ferdinandy et al., 2023). Controlled occlusions or pharmacological interventions activate a molecular self-defense program which delays the progression of infarction. The subsequent activation of multiple endogenous cardioprotective mechanisms including adenosine, protein kinase, calcium signalling, nitric oxide release, reduction in ROS but also the involvement of autacoids, hormones, neurotransmitters and cytokines lead to cardioprotection and reduce infarct size (Penna et al., 2022; Heusch, 2023).

The endocannabinoid system is suggested to play a role in preconditioning-induced cardioprotection as well. Importantly, its involvement was confirmed in different models of preconditioning (Table 2): ischemic preconditioning (Bouchard et al., 2003), remote preconditioning (Hajrasouliha et al., 2008), propofol- (Sun et al., 2017), NO- (Wagner et al., 2006), heat stress- (Joyeux et al., 2002) and LPS-induced preconditioning (Lamontagne et al., 2006) that led to the reduction in the infarct size (Lagneux and Lamontagne, 2001; Joyeux et al., 2002; Wagner et al., 2006; Sun et al., 2007; Hajrasouliha et al., 2008). Moreover, they were associated with prevention of ischemia-induced endothelial dysfunction (Bouchard et al., 2003), anti-oxidative (Sun et al., 2017), anti-arrhythmic effects (Hajrasouliha et al., 2008) and an improvement in cardiac contractility (Wagner et al., 2006).

The involvement of ECBs in the above studies was confirmed by three approaches (Table 2). Firstly, propofol- (Sun et al., 2017) and NO-preconditioning (Wagner et al., 2006) enhanced the release of AEA and 2-AG in heart and serum in *in vivo* studies in mice after LAD occlusion and in isolated neonatal rat ventricle cells exposed to hypoxia (Sun et al., 2017) and of 2-AG in perfused rat heart (Wagner et al., 2006). Secondly, the beneficial effect of propofol was mimicked by the FAAH inhibitor URB597 and the selective endocannabinoid reuptake inhibitor VDM11 (Sun et al., 2017), drugs that increase endocannabinoid levels, and the effect of ischemic preconditioning (Bouchard et al., 2003) was also obtained with 2-AG and palmitoylethanolamide (PEA). Moreover, 2-AG and its metabolically stable derivative noladinether mimicked the cardioprotective effects of NO-induced preconditioning (Wagner et al., 2006). Thirdly, the beneficial effect of preconditioning and ECBs was reversible and blocked mainly by the CB_2_R antagonists SR144528 and AM630 (Lagneux and Lamontagne, 2001; Joyeux et al., 2002; Bouchard et al., 2003; Sun et al., 2017). However, a contribution of CB_1_Rs is also suggested due to the blockade of beneficial effects by AM251 (Wagner et al., 2006) or rimonabant (Bouchard et al., 2003).

## 6 Cardioprotection in experimental myocardial ischemia/reperusion injury and acute myocardial infarction

### 6.1 Endocannabinoids

As described in the two previous chapters, alterations (mostly increases) in the levels of the endocannabinoids AEA and 2-AG occurred in humans suffering from coronary heart disease (Table 1) and in animals subjected to preconditioning prior to experimental cardiac ischemia (Table 2). Although the endocannabinoids may just accompany ischemia, some of the above studies suggest that they contribute to the pathophysiological events or serve as a counteracting system. There is a big body of studies on experimental animals *in vivo* (Tables 3, 4) and *in vitro* (Table 5) which provides additional evidence that alterations of endocannabinoid levels and the cellular mechanisms involved in ischemia are associated. In order to get an idea which role the endogenously formed endocannabinoids play, experiments with selective cannabinoid receptor antagonists, inhibitors of endocannabinoid degradation and knockout animals are interesting. In the second subsection, the question will be addressed how exogenously added endocannabinoids like AEA and 2-AG influence ischemia *in vivo* and *in vitro*.

#### 6.1.1 Effects of endogenously formed endocannabinoids?

In order to detect the possible *functional* role of either type of receptor, the use of selective CB_1_R and CB_2_R *antagonists* is a splendid technique. One can expect that the available antagonists (Figure 1) will cause an effect opposite in direction to that of the endocannabinoid(s). Even if the receptors are not exposed to an increased endocannabinoid level but are constitutively active, drugs like rimonabant or AM630 will reveal an endogenous tone since they are also inverse agonists (Maccarrone et al., 2023).

Some studies with CB_1_R antagonists suggest that CB_1_R activation has a detrimental influence. Thus, when the CB_1_R antagonist rimonabant was given once a day for 7 days before I/R, infarct size was reduced in wild-type CB_1_^+/+^ but not in CB_1_^−/–^ mice (Lim et al., 2009). Moreover, its administration for 7 days before or 6 h after AMI and its continuous use for 6 weeks improved cardiac contractility (by increasing the rate of developed left ventricle (LV) pressure) and reduced arterial stiffness and cardiac remodeling; preventive chronic treatment with rimonabant was more effective in comparison to post-ischemic administration (Slavic et al., 2013). On the other hand, AM251, another CB_1_R antagonist, which was started 24 h after AMI and continued for 12 weeks, failed to affect infarct size but promoted cardiac remodeling (Wagner et al., 2003). The same drug, however, reduced the damage of murine cardiomyocytes undergoing hypoxia by decreasing apoptosis, oxidative stress and mitochondrial injury (Hu et al., 2023). Incubation of murine atrial cardiomyocytes with rimonabant failed to inhibit cell death during H/R (Lim et al., 2009). Note that in most of the studies of Tables 4, 5 the effect of CB_1_R antagonists, given alone, was not examined.

Only one CB_2_R antagonist-based study suggests that endogenous CB_2_R activation has a beneficial effect. Thus, single administration of the CB_2_R antagonist AM630 before ischemia increased the infarct size and oxidative and inflammation parameters in anaesthetized mice (Di Filippo et al., 2004). Note that in most of the publications, CB_1_R and CB_2_R antagonists did not modify cardiac parameters by themselves (Tables 3–5).

The use of inhibitors of the degradation of endocannabinoids, e.g., of URB597, an inhibitor of the AEA-degrading enzyme FAAH, or of JZL184, an inhibitor of the 2-AG-degrading enzyme MAGL (Figure 1), represents another means to identify an interplay between endocannabinoids and mechanisms involved in ischemia. One has, however, to consider that the endocannabinoid level reached under blockade of the degrading enzyme may not reflect the true level occurring under ischemia. The experiments by Schloss et al. (2019) show that higher plasma and cardiac levels of 2-AG occur in mice after AMI and that 2-AG has an unfavorable CB2R-mediated effect in I/R and AMI in mice. Administration of JZL184 further enhanced cardiac neutrophil and monocyte recruitment and inflammation, enhanced infarct size, impaired ventricular remodeling, increased ventricular fibrosis and finally increased mortality (Table 3).

Knockout mice represent a third approach to identify a functional role of endocannabinoids in ischemia and AMI. Studies were performed with CB_2_^−^/^–^, FAAH^−/−^ and GPR55^−/−^ mice (GPR55 is an orphan G protein-coupled receptor activated by endocannabinoids (Guerrero-Alba et al., 2019; Puhl et al., 2021). One has to admit that knockout mice have the disadvantage that compensatory alterations may occur due to the life-long absence of a receptor or an enzyme.

CB_2_R deficiency in mice subjected to ischemia/reperfusion injury or AMI led to detrimental cardiac effects such as worse prognosis of cardiac infarction and profound I/R injury as heralded by an increase in infarct size, apoptosis and remodeling, fibrosis, collagen deposition, altered systolic and diastolic function and decreases in ejection fraction (EF), fractional shortening (FS) and stroke work (Defer et al., 2009; Duerr et al., 2014; 2015; Hu et al., 2019). These changes were accompanied by an increase in macrophage and neutrophil infiltrations, a decrease in anti-oxidative enzymes (Duerr et al., 2015), impaired cardiac work and a deterioration of the autophagy process (Hu et al., 2019) (Table 3). A similar picture emerged on cardiac cells isolated from CB_2_^−^/^–^ mice undergoing hypoxia (Table 5). Like in the *in vivo* experiments, one can observe detrimental cardiac effects such as the lack of decrease in levels of anti-oxidative enzymes and pro-autophagy proteins with a concomitant increase of LDH release, production of apoptotic factors and loss of cardiomyocytes (Duerr et al., 2014; 2015; Heinemann et al., 2015; Hu et al., 2019). Summarizing, all above experiments on knockout CB_2_^−^/^–^ mice clearly suggest the cardioprotective role of CB_2_R against I/R injury.

In FAAH-deficient mice, cardiac work after I/R deteriorated and manifested with an increase in fibrosis, left ventricle and cardiomyocyte hypertrophy, wall thickening and a decrease in fractional shortening (Rajlic et al., 2022), Table 4]. These detrimental cardiac effects evoked by enhanced endocannabinoid tone were reversed by the peroxisome proliferator-activated receptor α (PPARα) antagonist GW6471 suggesting the involvement of PPARα receptors [(Rajlic et al., 2022) Table 4].

Finally, GPR55 deficiency had no influence on infarct size in mice *in vivo* (Puhl et al., 2021). Nonetheless, GPR55 receptors had favourable effects on left ventricular load, compensatory hypertrophy, wound healing and maladaptation after AMI in that study (Table 4).

#### 6.1.2 Effects of exogenously administered endocannabinoids

Unfortunately, there are only few publications in which the effects of exogenously added AEA and 2-AG were examined in experimental models of I/R, AMI and hypoxia (Table 3-5). Thus, in rats with LAD occlusion AEA and/or its stable analogue methanandamide (MetAEA) reduced infarct size (Li et al., 2013a) and had an antiarrhythmic effect by improving myocardial resistance to arrhythmias induced by I/R (Krylatov et al., 2002). Similarly, in experiments on the isolated rat heart, 2-AG, PEA (Lépicier et al., 2003), AEA and MethAEA (Undertown et al., 2005) diminished infarct size and 2-AG and PEA improved cardiac work and had a beneficial effect against cardiac injury, as reflected by a decrease in lactate dehydrogenase (LDH) and creatine kinase (CK) levels in the perfusate (Lépicier et al., 2003).

The interaction of the endogenous cannabinoids with selective cannabinoid receptor antagonists was studied in three of the latter four studies (Tables 3–5). Thus, the infarct-limiting effect of AEA was antagonized by a CB_2_R antagonist (AM630 or SR144528) in the studies of Li et al. (2013a) and Undertown et al. (2005) whereas a CB_1_R antagonist (AM251 or RIM) had an antagonistic effect in the study of Undertown et al. (2005) only. In the study of Lépicier et al. (2003), the infarct-limiting effect of 2-AG and PEA was fully blocked by the CB_2_R antagonist SR144528 and the effect of 2-AG was also partially blocked by the CB_1_R antagonist RIM.

### 6.2 Synthetic cannabinoids

#### 6.2.1 Cardioprotection via cannabinoid CB_2_ receptors

As mentioned above, some of the studies described in Table 2 and experiments with rimonabant and CB_2_R-deficient mice (Tables 3, 5) suggest a cardioprotective role of CB_2_R activation. This view is further corroborated by *in vivo* experiments with animals exposed to I/R injury and treated with selective and unselective agonists of CB_2_Rs (Table 3). The latter agonists reduced infarct size, which is the most robust endpoint of cardioprotection studies in experimental models of myocardial ischemia. Thus, as shown in Table 3, AM1241 (Liu et al., 2021), JWH133 (Defer et al., 2009; Montecucco et al., 2009; Li et al., 2013b), HU308 (Wang et al., 2012) and WIN55,515-2 (Di Filippo et al., 2004) diminished infarct size in I/R models. Similarly, a limitation of infarct size was observed after administration of JWH133 (Yu et al., 2019) and HU308 (Wang et al., 2012) after AMI. One should keep in mind, that myocardial infarction is properly expressed as percentage of the area at risk which represents the myocardial perfusion bed distal to an occluded artery (Gimelli and Rovai, 2013; Heusch, 2019). Unfortunately, the reduction in infarct size in cannabinoid studies (Tables 3-5) was not correlated with the reduction of the area at risk (with one exception in the study of Liu et al. (2021). Additionally, Wang et al. (2012) expressed myocardial infarct size as a percentage of the infarct area over total LV area.

In addition to the reduction in infarct size, CB_2_R agonists showed other cardioprotective effects. Thus, an anti-fibrotic effect associated with a decrease in cardiac collagen content, fibronectin and other factors implicated in the fibrotic response to injury was demonstrated in the studies of Liu et al. (2021) and Wang et al. (2014). Anti-inflammatory effects were noticed as well and correlated with a reduction in inflammatory cell infiltration (Montecucco et al., 2009; Liu et al., 2021), serum and cardiac inflammatory cytokines (Wang et al., 2012; Wang et al., 2014; Li et al., 2016; Yu et al., 2019) and the level of the NLRP3 inflammasome [which controls proinflammatory processes; (Yu et al., 2019);]. Another beneficial consequence of CB_2_R activation was the decrease in oxidative stress (Montecucco et al., 2009; Wang et al., 2012; Wang et al., 2014; Li et al., 2016), the reduction in apoptosis (Li et al., 2013b; Liu et al., 2021) and the augmentation of autophagy (Liu et al., 2021). The above effects were closely correlated with the decrease in enzymes reflecting cardiac injury like serum troponin and creatine kinase (Montecucco et al., 2009; Yu et al., 2019; Liu et al., 2021) and the improvement in cardiac work (Wang et al., 2014; Li et al., 2016).

The involvement of CB_2_Rs in the cardioprotective effects of synthetic cannabinoids against I/R and AMI was confirmed in experiments with CB_2_R antagonists (Table 3). Thus, the beneficial cardiac effects of JWH133 (Montecucco et al., 2009; Li et al., 2013b; Yu et al., 2019) and HU308 (Wang et al., 2012) were diminished by the CB_2_R antagonist AM630. The use of CB_2_R antagonists also allowed to decide that compounds possessing affinity for both CB_1_Rs and CB_2_Rs [WIN55,212-2; (Di Filippo et al., 2004; González et al., 2011); and AEA; (Li et al., 2013b);] act through CB_2_Rs since the improvement in cardiac work and coronary pressure and the decrease in infarct size and inflammatory responses induced by unselective agonists were attenuated or abolished by AM630 but not by the CB_1_R antagonist AM251. In this context, also the beneficial effect of curcumin, which reduced biochemical markers of cardiac injury, oxidative stress and inflammation against AMI in mice with streptozotocin-induced diabetes mellitus should be mentioned. Its effects were antagonized by AM630 (Pawar et al., 2022); CB_1_R antagonist not studied]. Curcumin is a polyphenol derived from the perennial plant *Curcuma longa*, which interacts with CB_2_Rs as shown in molecular docking studies (Pawar et al., 2022). A meta-analysis of 37 preclinical studies involving 771 rats or mice confirmed recently that curcumin exerts an excellent potential for the treatment of myocardial I/R injury in animal models. Surprisingly, the authors did not mention cannabinoid receptors in their publication at all (Zeng et al., 2023).

Cardioprotective effects of CB_2_R agonists against I/R injury have also been shown *in vitro* in cardiac cells and in isolated heart preparations subjected to hypoxia and ischemia, respectively (Table 5). In rat cardiomyocytes undergoing hypoxia with subsequent reoxygenation, incubation of cells with AM1241 (Liu et al., 2021) had a beneficial cardiac influence against H/R injury; so, it increased the production of pro-autophagy-related proteins and decreased the production of collagen, other pro-fibrotic factors and ROS (Li et al., 2016). In murine cardiomyocytes JWH133 increased cell survival and diminished hypoxia-dependent increase in the NLRP3 inflammasome (Yu et al., 2019). Moreover, in studies on isolated rat heart, JWH133, JWH015 and AM1241 decreased infarct size and improved cardiac recovery and work in hearts subjected to LAD occlusion (Li et al., 2014) or to a low-flow protocol (Lépicier et al., 2003; 2006; 2007). Only in the study by Undertown et al. (2005), JWH133 did not diminish infarct size although a 10-fold higher concentration was used when compared to the above studies. The authors started heart perfusion with JWH133 only 5 min before ischemia (Undertown et al., 2005); this time interval had proven to be sufficient for demonstration of the beneficial influence of JWH015 (Lépicier et al., 2007), AEA and MethAEA (Undertown et al., 2005) (Table 5). The results with JWH133 are surprising since, as mentioned above, AEA showed an infarct-limiting effect in the study of Undertown et al. (2005).

Final proof for the involvement of CB_2_Rs in the cardioprotection against I/R injury *in vitro* was obtained by the use of CB_2_R antagonists (Table 5). Thus, AM630 and/or SR144528 diminished the beneficial effects of JWH133 and/or JWH015 in the isolated rat heart, respectively (Lépicier et al., 2007; Li et al., 2014).

The results obtained with the nonselective agonist HU210 shall be discussed separately. HU210 decreased the weight of the necrotic zone without affecting hypoperfused area flow in rats after I/R (Ugdyzhekova et al., 2002), improved cardiac work recovery and decreased infarct size, area at risk and cardiac injury (visible as a decrease in the level of creatinine kinase in the perfusate) after I/R (Maslov et al., 2006; Gorbunov et al., 2016). Unfortunately, in none of the latter studies, the exact type(s) of cannabinoid receptor(s) has been determined. In the study of Wagner et al. (2003), twelve week-administration of this agonist started 24 h after AMI failed to affect infarct size and mortality in rats but improved cardiac contractility and prevented endothelial dysfunction in aortic rings and hypotension whereas the CB_1_R antagonist AM251 promoted cardiac remodeling. One has to consider that the fact that HU210 had a cardioprotective and AM251 a detrimental effect (Wagner et al., 2003) does not prove that HU210 acted via CB_1_Rs; again, interaction experiments with a CB_2_R antagonist have not been performed.

The possibility that CB_2_Rs, e.g., activated by HU-210, interact with β_1_-adrenoceptors (β-ARs) that play an important role in the regulation of cardiac tolerance to ischemia and reperfusion (e.g., Maslov et al., 2024) had to be considered. Interestingly, HU-210 and WIN55212-2 diminished the positive inotropic and chronotropic effects of the non-selective β-AR agonist, isoprenaline, and reduced the isoprenaline-stimulated increase in cAMP formation in isolated rat hearts (Maslov et al., 2004) and neonatal cardiomyocytes (Liao et al., 2013). Regarding cardiac ischemia-mediated injury, only one study on rats has been carried out, which reveals that the chronic administration of β-caryophyllene (BCP), a naturally occurring dietary cannabinoid (50 mg/kg, orally; twice daily for 10 days), diminished the isoprenaline-induced myocardial injury (including impaired cardiac function, increased levels of serum cardiac marker enzymes, and enhanced oxidative stress). In that study, isoprenaline (85 mg/kg) was given at an interval of 24 h for 2 days (ninth and 10th day) and its effect was partially sensitive to the CB_2_R antagonist AM630 (1 mg/kg given i. p. chronically prior to BCP treatment for 10 days) (Meeran et al., 2021).

Unfortunately, the combination of a CB_2_R agonist and a β-AR *antagonist* has so far not been studied in an animal model of cardiac ischemia-mediated injury. One should keep in mind that acute and chronic treatment with β-AR antagonists is frequently used to improve the outcome of the acute (Giannakopoulos and Noble, 2021) and chronic (Kim et al., 2020) phase of acute myocardial infarction in humans. It would be interesting to know whether the beneficial effect of β-AR antagonists (which block a G_s_ protein-coupled receptor) can be further increased by a CB_2_R agonist (which activates a G_i_ protein-coupled receptor).

#### 6.2.2 Cardioprotection via other receptors/mechanisms

The putative involvement of other types of receptors, and in particular of CB_1_Rs, in the cardioprotective effects of synthetic cannabinoids during ischemia has been demonstrated in a few publications *in vivo* (Table 4) and *in vitro* (Table 5). The results regarding the role of CB_1_Rs in I/R injury are inconsistent. It has already been mentioned above that studies with CB_1_R antagonists suggest that CB_1_Rs have a beneficial (Lim et al., 2009)–*in vivo* experiments only; (Wagner et al., 2003; Slavic et al., 2013);] or detrimental effect (Hu et al., 2023) or no effect at all [(Lim et al., 2009)–*in vitro* experiments only)].

In one study, a synthetic selective CB_1_R agonist has been administered. Arachidonyl-2-chloroethylamide (ACEA) diminished infarct size in isolated rat heart in a manner sensitive to the CB_1_R antagonist rimonabant (Lépicier et al., 2007).

Apart from CB_2_Rs and CB_1_Rs, other receptors may come into play during I/R. As mentioned above, experiments with GPR55-deficient mice suggest that this receptor [activated by endocannabinoids but mainly by the endogenous agonist L-α-lysophosphatidylinositol, LPI; (Puhl, 2020);] has cardioprotective properties (Puhl et al., 2021) (Table 4). In another study on GPR55-deficient mice, Robertson-Gray et al. (2019) showed that LPI administration before ischemia (but not reperfusion) increased infarct size in the perfused heart of wild-type mice but not in mice with deletion of GPR55 receptors (Table 5). A satisfactory explanation for the discrepancy between the studies of Puhl et al. (2021) and Robertson-Gray et al. (2019) cannot be given.

### 6.3 Phytocannabinoids

Although *Cannabis sativa* contains numerous cannabinoids, Δ^9^-tetrahydrocannabinol and cannabidiol prevail. Their potential cardioprotective effects were examined in a series of studies.

The use of *Δ* ^*9*^ *-tetrahydrocannabino*l (THC) in cardiac I/R injury has been controversial and is strongly limited by its psychoactive properties (Leinen et al., 2023) (Table 4, 5). An ultra-low dose of THC (0.002 mg/kg, intraperitoneally) decreased both infarct size (given as a single bolus and chronically for 3 weeks before AMI) and cardiac damage in mice treated with this agent before AMI (Waldman et al., 2013). In line with the above report are the results of Shmist et al. (Shmist et al., 2006) who found that a 24 h incubation of rat cardiomyocytes undergoing hypoxia with THC protected cells from injury in an NO-dependent manner. Additionally (Banaszkiewicz et al., 2022), incubation of murine cardiomyocytes with THC under chemical hypoxia increased cardiomyocyte contractility and cytoplasmic LDH activity. Importantly, in human cardiomyocytes subjected to chemical hypoxia THC also exerted cardioprotective effects related to an improvement in cell metabolism and antioxidative activity, mitochondrial protection and a decrease in cell mortality. Moreover, THC improved recovery of the isolated rat heart after I/R.

The CB_2_R antagonist SR144528 blocked the beneficial effects of THC in rat cardiomyocytes whereas the CB_1_R antagonist RIM failed to do so (Shmist et al., 2006). Unfortunately, the cannabinoid receptor(s) involved in the cardioprotective actions of THC *in vivo* (Waldman et al., 2013) and *in vitro* (Banaszkiewicz et al., 2022 has/have not been determined.

The non-intoxicating and well-tolerated multitarget *cannabidiol* (CBD) possesses a great therapeutic potential resulting from its strong anti-inflammatory, anti-oxidant and anticonvulsant properties (Atalay et al., 2019; Kicman and Toczek, 2020; Leinen et al., 2023). Few studies also suggest potential cardioprotective properties against I/R (Table 4; 5). Thus, chronic treatment with CBD diminished infarct size in experiments with cardiac I/R in rats and rabbits (Table 4) (Durst et al., 2007; Garberg et al., 2017). Interestingly, two single injections, given before I and R, were also effective in decreasing the infarct size (Walsh et al., 2010; Feng et al., 2015). By contrast, CBD given intraperitoneally in two single boluses to rats with subsequent I/R studied on the isolated perfused heart, failed to decrease the infarct size; this may suggest that CBD does not have an equivalent effect *in vivo* and *in vitro* and that complex systemic mechanisms are responsible for the positive CBD effect *in vivo* (Durst et al., 2007). Other beneficial effects of CBD in I/R studies include improvement in cardiac work in the isolated heart (Franco-Vadillo et al., 2021) and in experiments *in vivo* (Durst et al., 2007; Feng et al., 2015; Franco-Vadillo et al., 2021), anti-inflammatory (Durst et al., 2007; Feng et al., 2015) and antiarrhythmic effects (Walsh et al., 2010; Gonca and Darıcı, 2015) as well as inhibition of platelet aggregation (Walsh et al., 2010). In addition, CBD decreased the necrotic zone (but not the area at risk), and increased the blood flow in the area at risk (Durst et al., 2007; Feng et al., 2015) (Table 4).

The mechanisms responsible for the cardioprotective effects of CBD may include a reduction in inflammatory responses (Durst et al., 2007; Feng et al., 2015), modulation of the angiotensin-renin system (increase in the expression of AT_2_ receptors and prevention of an increase in AT_1_ receptors responsible for, e.g., vasoconstriction, inflammation and remodeling) and RISK pathway stimulation (Franco-Vadillo et al., 2021). The antiarrhythmic potency of CBD in I/R injury is due to adenosine A_1_ receptor activation (Gonca and Darıcı, 2015).

## 7 Potential cellular mechanisms of cardioprotective actions of cannabinoids in ischemia/reperfusion injury

It was not the purpose of this review to analyze the cellular mechanisms of cardioprotective cannabinoids (beyond the level of receptors) and for this reason they are summarized here only briefly. Tables 1-5 show that the potential cellular pathways of cardioprotective cannabinoids have been examined in few studies only.

Several recent reviews have highlighted cardioprotective mechanisms that can regulate mitochondrial function, autophagy processes, modulation of energy metabolism, inflammation and apoptosis, and protect from ischemia-reperfusion injury (Ferdinandy et al., 2023). They include activation of PI3K (phosphoinositide-3-kinase (PI3K) and Akt (protein kinase B) (Deng and Zhou, 2023), ERK1/2 (extracellular signal-regulated kinase) (Kong et al., 2019), Pink1/Parkin (PTEN-induced kinase 1 (PINK1) and the E3 ubiquitin ligase Parkin) (Yu and Miyamoto, 2021), AMPK (AMP-activated protein kinase) (Pakesh et al., 2022), p38 MAPK (p38 mitogen-activated protein kinase) (Ruiz et al., 2018) and TGF-β (transforming growth factor-β1)-dependent activation of Smad-dependent cascades (Humeres et al., 2022).

Regarding the cardioprotective effects of CB_2_R stimulation against I/R injury, the possible involvement of the pro-survival PI3K/Akt pathway (Li et al., 2013a; 2013b; Wang et al., 2014), the ERK1 pathway (Montecucco et al., 2009) and induction of autophagy via the Pink1/Parkin pathway (Liu et al., 2021) was shown. Moreover, in mice with deletion of CB_2_Rs deterioration of the autophagy process related to inhibition of cardiac protective AMPK signaling was described (Hu et al., 2019). In an *in vitro* study, the anti-fibrotic and anti-oxidative actions of CB_2_R activation leading to the inhibition of the TGF-β1/Smad3 pathway was shown (Li et al., 2016). Blockade of CB_1_Rs with AM251 reduced the damage of cardiomyocytes exposed to hypoxia by activation of the AMPK pathway (Hu et al., 2023). With respect to the cannabidiol-dependent cardioprotective actions against I/R injury, the PI3K/Akt and MAPK/ERK pathways were shown as the only cellular mechanisms (Franco-Vadillo et al., 2021). Conversely, the enhancement of cardiac injury during ischemia mediated via activation of GPR55 receptors was dependent on the activation of the ROCK/p38 MAPK (Rho-assisted protein kinase/p38 mitogen-activated protein kinase) pathway (Robertson-Gray et al., 2019). Consequently, further research is necessary to determine the exact cellular signaling pathways by which cannabinoids produce beneficial (or detrimental) effects on the heart.

Little information is so far available with respect to the cellular sources of ECBs during I/R or AMI. Endocannabinoids and enzymes involved in their synthesis were found in rat cardiac tissues, endothelial cells and macrophage-derived cell lines, which means that cardiac function and coronary perfusion might be modulated by endocannabinoids derived not only from cardiac tissues but also from circulating cells like macrophages and platelets (Pacher and Haskó, 2008; Puhl, 2020).

## 8 From preclinical studies to clinical settings–IMPACT criteria

Various drugs or surgical interventions have been shown to reduce infarct size and to improve cardiac function and healing in preclinical studies but despite the optimistic results none of the drugs/interventions could be implemented for preventing myocardial ischemia/reperfusion injury in patients with AMI (Bolli, 2021; Heusch, 2023). Promising cardioprotective effects have also been found in preclinical studies of cannabinoids including mainly CB_2_R agonists and the multitarget CBD (for review, see (Lamontagne et al., 2006; Pacher and Haskó, 2008; Steffens and Pacher, 2012; Maslov et al., 2016; Tang et al., 2021) but drugs for the use in humans have so far not been developed. The question arises, why successful preclinical results for the treatment of acute myocardial infarction do not translate well into clinical practice (Heusch, 2023)?

**FIGURE 3:**
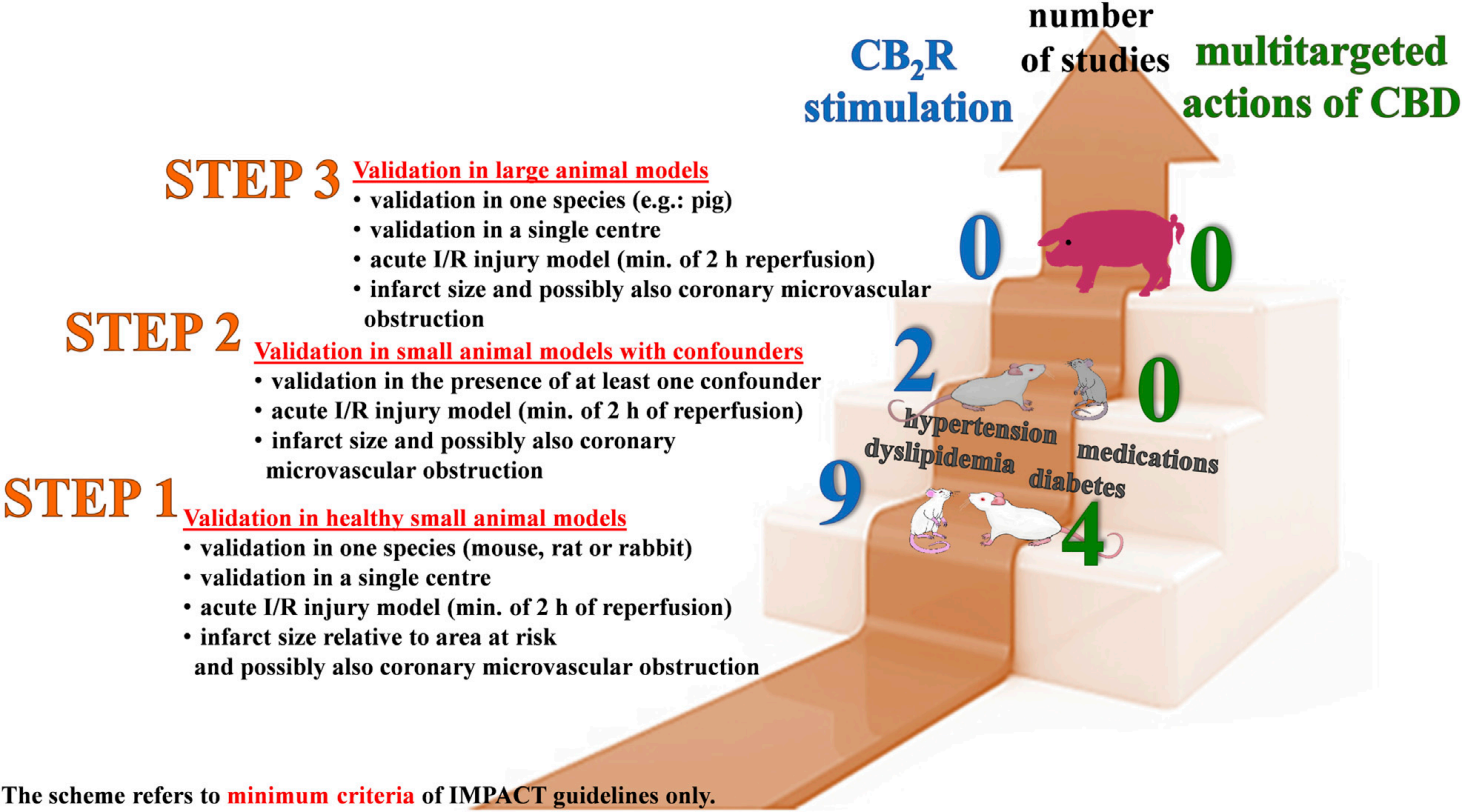
Are the studies regarding the cardioprotective effects of cannabinoid CB_2_ receptor (CB_2_R) agonistic drugs and cannabidiol (CBD) against cardiac ischemia/reperfusion (I/R) in accordance with the minimum (min.) criteria of the IMPACT (IMproving Preclinical Assessment of Cardioprotective Therapies) three-step schedule proposed by the European Union (EU) CARDIOPROTECTION COST ACTION (Lecour et al., 2021)? Our literature review, which is the basis for this scheme, shows that only 9 (blue numbers) and 4 (green numbers) of the studies examining the effects of CB_2_R activation and CBD, respectively, meet the minimum criteria of STEP 1. The minimum criteria of STEP 2 are partially met by two studies with CB_2_R activation only. Studies in large animals (STEP 3) are still lacking. For clarification: to meet STEP 2, experiments should be performed on small animals with con-founders such as co-morbidities (e.g., diabetes mellitus) or medications (used during surgery or for treatment of diseases) since these factors may influence the observed results.

To solve the above problem, the European Union (EU) CARDIOPROTECTION COST ACTION published in 2021 gives step-by-step criteria for Improving Preclinical Assessment of Cardioprotective Therapies (IMPACT) that should be met to improve the likelihood of translating novel cardioprotective interventions into the clinical setting (Lecour et al., 2021). They consist of three steps and their minimum criteria are given here (Figure 3). STEP 1 means that experiments should be validated in one species of small animals (e.g., mouse, rat or rabbit) in a single centre in an acute I/R injury model (minimum of 2 h but preferably 24 h of reperfusion). The end-point of the study should be the measurement of infarct size relative to area at risk and possibly also coronary microvascular obstruction. STEP 2 requires validation in small animal models in the presence of at least one confounder since a variety of factors (age, sex, diabetes, hypertension, dyslipidemia) and co-medications (platelet inhibitors, anesthetics, anti-diabetic drugs, statins and nitrates) might interfere with the end-points of the treatment. STEP 3 includes criteria for validation in large animal models. The importance of experiments on small animals with comorbidities and co-medications as well as on large animals have recently been underlined also by other groups (Lindsey et al., 2021; Penna et al., 2022; Ferdinandy et al., 2023; Wang et al., 2023).

Since IMPACT accepts *in vivo* preclinical studies only, we have checked the studies regarding cardioprotective effects of CB_2_R activation and collected in Table 3. Only nine studies met the criteria of STEP 1 (Di Filippo et al., 2004; Defer et al., 2009; Montecucco et al., 2009; Wang et al., 2012; Li et al., 2013a; 2013b; Duerr et al., 2014; 2015; Liu et al., 2023) The minimum criteria of STEP 2 were partially met by two studies only. Although the latter studies were performed on diabetic and/or fatty rats after I/R (González et al., 2011) or mice after AMI (Pawar et al., 2022) changes in infarct size were not determined. Finally, studies in large animals (STEP 3) are still lacking (Figure 3).

The EU CARDIOPROTECTION COST ACTION also stresses the importance of multitarget therapies as effective tools against myocardial ischemia/reperfusion injury (Davidson et al., 2019) With respect to the multitarget CBD we have found that only four among the seven papers in Table 4 met the minimum criteria of STEP 1 (Durst et al., 2007; Walsh et al., 2010; Feng et al., 2015). Studies on small animals with comorbidities or on large animals (STEP 2 and 3, respectively) have not been performed at all (Figure 3). The article by Garberg et al. (2016) on newborn piglets undergoing global hypoxia and treated with a high dose of CBD could not be considered in our analysis since cardiac ischemia was not quantified directly in that paper.

Apart from CBD and from drugs leading to CB2 receptor activation, only the study by Lim et al. (2009), dedicated to the cardioprotector effects of the CB_1_ receptor antagonist rimonabant, meets the criteria of STEP 1 and STEP 2 (Table 4).

Most of the studies in which potential cardioprotective properties of cannabinoids against I/R injury and AMI were examined took place before the IMPACT criteria were established. So, the experiments were conducted under experimental protocols in which reperfusion was not examined or, if so, was not long enough. Furthermore, compounds were administered prior to the onset of ischemia and the area of infarction was not always assessed in relation to the area at risk. Thus, the putative cardioprotective properties of cannabinoids against I/R and AMI should be re-evaluated in experiments on small animals under appropriate conditions and on large animals.

## 9 Limitations

Our literature review reveals that the protocols used to induce ischemia/hypoxia and the compounds under study extremely varied. Furthermore, most experiments were conducted on healthy rodents and cannabinoids were administered once only. Therefore, we were unable to give details to (1) the best timing of (endo)cannabinoid administration, i.e., before, during or after ischemia (from the clinical perspective, the best time for treatment is after ischemia or AMI, but this time of administration had been chosen by few of the studies only); (2) the effect of (endo)cannabinoids in animals with comorbidities and under chronic drug treatment; (3) the detrimental effects of the blockade of endocannabinoid degradation since only few studies of that type had been performed (Schloss et al., 2019; Rajlic et al., 2022). Moreover, the cardioprotective effects of (endo)cannabinoids in the human heart had been examined in few studies only (Table 1). Finally, in the light of the fact that the major heart receptors, the β-ARs, are essential for adjusting the heart to ischemia-reperfusion injury, it is unsatisfactory that the cross-talk between these receptors and the cannabinoid receptors had been examined in one study only (Meeran et al., 2021).

## 10 Conclusion

A detailed review of all publications regarding cardiac effects of cannabinoids clearly shows that cannabinoids exert cardioprotective effects in cardiac preconditioning, I/R and AMI. Beneficial cardiac actions result mainly from the activation of CB_2_Rs although positive effects of the endocannabinoid AEA, the phytocannabinoid THC and the multitarget CBD were also described. Importantly, endocannabinoid levels were enhanced in acute and chronic cardiac disorders in humans including AMI.

In 2021 the strict step-by-step criteria for Improving Preclinical Assessement of Cardioprotective Therapies (IMPACT) have been established by the EU CARDIOPROTECTION COST ACTION (Lecour et al., 2021). In our evaluation of the papers regarding the cardioprotective effects of cannabinoids in the context of various protocols and models none of the publications so far met all IMPACT criteria. Thus, additional experiments are needed to confirm the cardioprotective activities of cannabinoids on small animals with comorbidities and on large animals. One should also keep in mind that it is necessary that the infarct size be expressed in relation to the area at risk. Additionally, as a result of our review of the literature, we have been able to identify the main directions in which intensive research should be conducted in order to gain a closer understanding of the cardioprotective effects of cannabinoids. Thus, the proper moment of administration of drugs (so far generally administered before ischemia, i.e., before the moment most difficult to predict for the patient) should be identified, since early reperfusion constitutes the critical time to perform life-saving interventions (Gibson, 2001; Heusch, 2020). Moreover, one should consider the use of a new generation of CB_2_Rs agonists (characterized, e.g., by a better solubility) that have shown promising results in preclinical studies related to analgesic and anti-inflammatory effects (Bryk and Starowicz, 2021; Whiting et al., 2022). With respect to the putative therapeutic application of FAAH or MAGL inhibitors (Toczek and Malinowska, 2018; Maccarrone et al., 2023) one should be particularly careful because chronic enhancement of the endocannabinoid tone caused detrimental cardiac effects in animal models (Schloss et al., 2019; Rajlic et al., 2022). Importantly, more new data regarding the cardiac endocannabinoid system in humans under pathological conditions are also needed.

Taken together, we believe that it is too early to place great hopes on the future cardioprotective application of cannabinoids (including CB_2_R agonists) in myocardial ischemia. Thus, tools and methods used for further studies must be well elaborated in order to meet the current shortcomings in our knowledge regarding the cardioprotective actions of cannabinoids against I/R injury.
